# Spectroscopic and Computational pH Study of Ni^II^ and Pd^II^ Pyrrole-Imine Chelates with Human Serum Albumin

**DOI:** 10.3390/molecules28227466

**Published:** 2023-11-07

**Authors:** Sheldon Sookai, Matthew Lee Bracken, Monika Nowakowska

**Affiliations:** Molecular Sciences Institute, School of Chemistry, University of the Witwatersrand, Johannesburg PO WITS 2050, South Africa; matthewbracken125@gmail.com (M.L.B.); monika.nowakowska@wits.ac.za (M.N.)

**Keywords:** albumin, ligand binding, DFT simulations, metal chelate, Schiff base chelate, circular dichroism, molecular dynamics, fluorescence

## Abstract

Human serum albumin (HSA) efficiently transports drugs in vivo: most are organic. Therefore, it is important to delineate the binding of small molecules to HSA. Here, for the first time, we show that HSA binding depends not only on the identity of the d^8^ metal ion, Ni^II^ or Pd^II^, of their complexes with bis(pyrrole-imine), H_2_PrPyrr, but on the pH level as well. Fluorescence quenching data for native and probe-bound HSA showed that sites close to Trp-214 (subdomain IIA) are targeted. The affinity constants, K*a*, ranged from ~3.5 × 10^3^ M^−1^ to ~1 × 10^6^ M^−1^ at 37 °C, following the order Pd(PrPyrr) > Ni(PrPyrr) at pH levels of 4 and 7; but Ni(PrPyrr) > Pd(PrPyrr) at a pH level of 9. Ligand uptake is enthalpically driven, dependent mainly on London dispersion forces. The induced CD spectra for the protein-bound ligands could be simulated by hybrid QM:MM TD-DFT methods, allowing us to delineate the binding site of the ligands and to prove that the metal chelates neither decompose nor demetallate after uptake by HSA. The transport and delivery of the metal chelates by HSA in vivo is therefore feasible.

## 1. Introduction

Pyrrole-based compounds have garnered substantial attention in scientific research due to their inherent bioactivity and prominent role in pharmaceutical applications [1,2]. Among these compounds, pyrrole-imine Schiff bases have been shown to have the ability to chelate a wide range of metal ions [3,4,5,6,7,8], including Ni^II^ [9,10,11] and Pd^II^ [12,13], leading to the formation of stable complexes characterized by metal-pyrrolide and metal-imine bonds following the concurrent deprotonation of the pyrrole NH group. Pyrrole compounds serve as essential building blocks in the development of organic pharmaceuticals [14]. Notable examples include Tolmetin [15], Sunitinib [16], and Glimepiride [17]. Studies conducted over the past two decades highlight the significant medicinal potential exhibited by pyrrole-imine metal chelates. One of the most well-known instances of such chelation is the pentadentate macrocycle known as texaphyrin [18], which complexes with lanthanides. These compounds, while not yet FDA-approved, hold significant promise for applications in photodynamic cancer chemotherapy [19] and MRI contrast applications [20], respectively. Despite these advancements, there remains a limited body of work focused on elucidating the binding mechanisms of these compounds to human serum albumin (HSA). This investigation is pivotal for a comprehensive understanding of the distribution of metal chelate drug candidates within the plasma [21]. In the present study, our primary objective was to employ simple pyrrole-imine metal chelates complexed of Ni^II^ and Pd^II^ to examine their interaction with HSA under varying pH conditions. After the emergence of drug-resistant cancers, transition metal complexes have been extensively investigated for their anti-proliferative activity. In particular, less expensive precious metal ions like Ni^II^ and Pd^II^ have garnered attention [22,23]. However, there is limited research on the interaction of Ni^II^ and Pd^II^ pyrrole Schiff base chelates with HSA. Our aim was to gain insight into how changes in pH, while maintaining a constant ionic strength, influences the binding of the chelates to the protein. Furthermore, we aimed to assess the potential role of HSA as a transporter or delivery vehicle for these compounds in vivo.

HSA is the most abundant serum protein, present in the blood at concentrations of ~600 µM [24]. Its primary functions are to maintain colloidal osmotic pressure and the transportation of both endogenous and exogenous compounds [25]. A notable characteristic of HSA lies in its remarkable capacity to interact with a broad spectrum of pharmaceutical compounds and steroids [26,27,28]. This interaction is a pivotal initial step in the processes of detoxification, solubilization, and distribution of these pharmaceutical compounds among various tissues [25]. Consequently, HSA plays a vital role in safeguarding bound drugs against oxidation or reduction. Given the substantial prevalence of HSA in plasma and the strength of its binding interactions with drugs, HSA emerges as a critical factor to consider in the design and development of novel medications [29,30,31]. The delineation of the binding mechanisms between medicinal compounds and HSA holds central importance in comprehending their pharmacodynamic and pharmacokinetic profiles in vivo. This is especially significant due to the abundance of HSA and its role as a transporter in blood plasma [32].

In the body, HSA is exposed to pH levels varying from 4.5 to 8; however, most studies measure the binding affinity of drugs to HSA under physiological conditions but do not consider a buffer’s ionic strength or the change in the pH level of different vesicles within the blood [33]. HSA is well documented to transition between pH levels; at a physiological pH level it takes on the N-form which is heart-shaped, and transitions to unfolded states at acidic and alkaline pH levels, respectively. At an acidic pH level, HSA transitions from the N to F form (pH levels of 5–3.5) and below a pH level of 3.5 it transitions to the E-form [34,35,36], while at an alkaline pH level, HSA transitions from the N to B form [37]. At each pH level, the different forms of HSA are able to bind to ligands with different affinities [38,39,40].

In vivo, pH regulation is essential for biochemical processes and is controlled by proton transfer [41]. Measuring the binding of small molecules to HSA at different pH levels is important for drug formulation and processing, as the pH level influences conformational changes in the protein. Furthermore, it is estimated that ~67% of drugs are ionizable [42]. The maintenance of pH levels is a crucial aspect of ensuring the effectiveness and quality of drug formulations. pH regulation plays a significant role in enhancing various thermodynamic aspects of reactions, promoting chemical stability, and improving solubility. In pharmaceutical processes like autoclaving, homogenization, stability testing, and storage, pH level emerges as a critical quality parameter in numerous systems and formulations [42].

The selection of an appropriate buffer is of paramount importance in elucidating the influence of pH levels on ligand binding to HSA [43]. The choice of buffer should align with the experimental conditions and possess a relevant p*K*_a_ value. However, a practical aspect often overlooked in pH-related studies is the control of ionic strength across different pH levels [43,44]. Ionic strength plays a crucial role since it reflects the ionic environment surrounding a protein, where the protonation/deprotonation of amino acid residues occurs at various pH values. This dynamic environment can potentially impact the affinity of proteins for ligands. Neglecting to control the ionic strength in pH variation studies may confound the interpretation of alterations in binding affinity at different pH values, as the observed differences may be attributed not only to the pH value but also to variations in the ionic strength. An effective approach to address this issue is the utilization of multi-component buffers capable of maintaining a constant ionic strength over a broad pH range [44].

**Figure 1:**
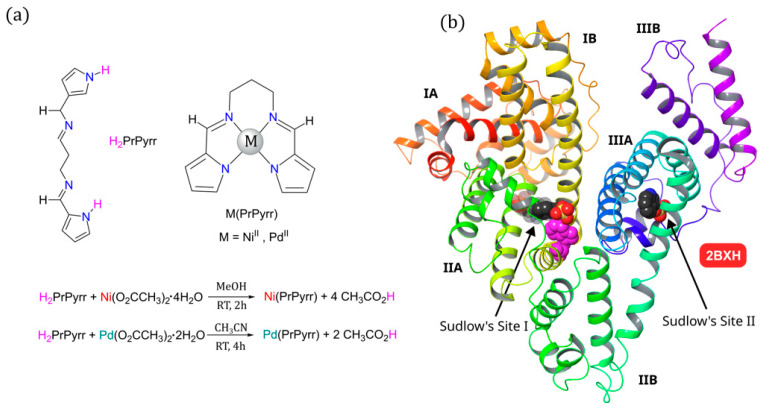
(**a**) Structures of the bis(pyrrole-imine) ligand H_2_PrPyrr (*N*,*N*′-bis [(1*E*)-1*H*-pyrrol-2-ylmethylene]propane-1,3-diamine) and its neutral Ni^II^ and Pd^II^ chelates with square planar d^8^ metal ions relevant to this work. The method for metalation of the ligand is illustrated for the synthesis of both Ni(PrPyrr) and Pd(PrPyrr). (**b**) X-ray structure of HSA bound to indoxyl sulfate (redrawn from PDB code 2BXH) [45] illustrating the two main small molecule binding sites. Indoxyl sulfate binds in a single mode to Site II while Site I can bind the drug in two orientations. Sudlow’s Site I is larger than Sudlow’s Site II and compounds that bind in this pocket perturb the fluorescence from Trp-214 (shown in magenta). The protein secondary structure elements are depicted schematically, colored by domain, and labeled with Roman numerals and Arabic letters.

In this study, we synthesized a widely recognized tetradentate bis(pyrrole-imine) ligand, H_2_PrPyrr, along with its isoelectronic (*n*d^8^) square-planar chelates of Ni^II^ and Pd^II^, denoted as M(PrPyrr) (Figure 1a). We employed spectroscopic techniques to investigate the binding interactions of H_2_PrPyrr and M(PrPyrr) with HSA across various pH values, all while ensuring a constant ionic strength. To achieve this, we employed a multicomponent acetate-MES-Tris buffer (AMT). Our goal was to assess how the presence of the metal ion influences the uptake of these complexes and to ascertain their preferred binding site(s) at different pH levels. For the first time, we provide comprehensive insights into the binding affinity, thermodynamics, and virtually all aspects of the physical biochemistry influenced by pH level (while maintaining a constant ionic strength) for *n*d^8^ square-planar chelates binding to HSA. Despite the inherent complexity of the system, our spectroscopic data are corroborated by advanced in silico methods, demonstrating good agreement. This study serves as a foundational template for the measurement of biophysical parameters related to drug binding to HSA and the validation of such data through in silico approaches.

## 2. Results and Discussion

### 2.1. Metal Chelate Synthesis

Modified literature methods were implemented to metalate the bis(pyrrolide-imine) chelate (H_2_PrPyrr) with Ni [22] and Pd [46,47]. The metal salts of choice in the reactions were Ni^II^ and Pd^II^ acetate, as depicted in the protocol in Figure 1a. The free acetate ions served as an adequate base to accept the pyrrole NH protons released upon the metalation of H_2_PrPyrr. Both metal Schiff base chelates were isolated cleanly and did not require any further purification.

### 2.2. pH Speciation of Ni(PrPyrr) Species

**Figure 2:**
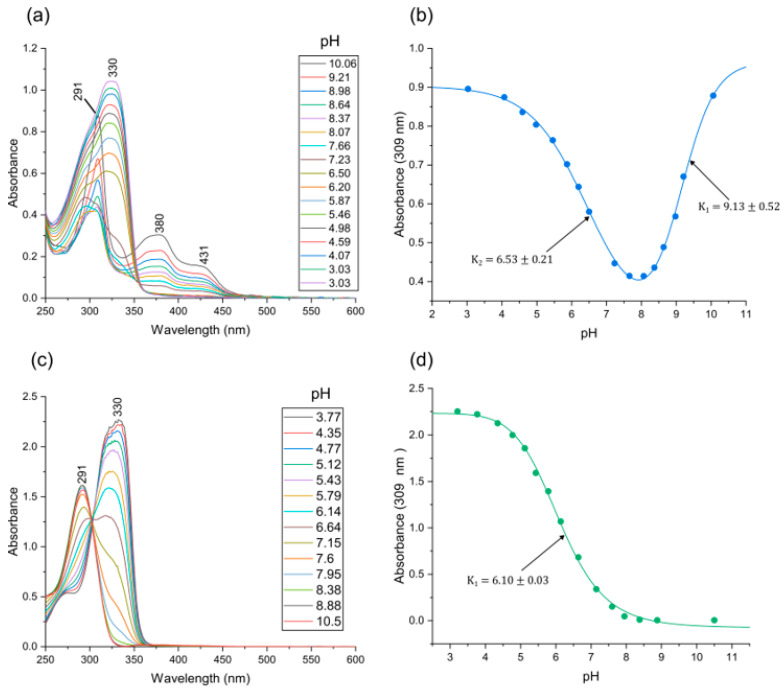
(**a**) UV-visible spectra of Ni(PrPyrr) recorded as a function of pH level at 298 K. (**b**) Plot of the change in absorbance at 309 nm as a function of pH level. The curve is a nonlinear fit of the data to a standard two-step p*K*_a_ equilibrium function (H_2_A ⇌ HA + H^+^, K_1_; HA ⇌ A + H+, K_2_) and had a R^2^ = 0.998. (**c**) UV-visible spectra of H_2_PrPyrr recorded as a function of pH level at 298 K. (**d**) Plot of the change in absorbance at 309 nm as a function of pH level. The curve is a nonlinear fit that is fitted to the Hill fit function of the data to a standard one-step p*K*_a_ equilibrium function) and had a R^2^ = 0.998.

In order to investigate the potential hydrolysis of Ni(PrPyrr), we conducted a series of UV-vis spectroscopic measurements on the Ni^II^ complex across a pH range of 10 to 3, closely monitoring alterations in the complex’s spectrum at 309 nm (Figure 2a). Upon initial examination, our data revealed a nonlinear bi-dose response fit, indicative of two distinct p*K* values occurring at a pH level of 9.13 (±0.5) and a pH level of 6.13 (±0.2) (Figure 2b). To provide a mechanistic understanding of the observed behaviour of Ni(PrPyrr) at each p*K* value, we present plausible reaction schemes in the equations below.

Ni(PrPyrr) + NaOH → No reaction (1)

Ni(PrPyrr) + 2H_2_O → H_2_PrPyrr + Ni(OH)_2_ (2)

Ni(PrPyrr) + 3HCl → [H_3_PrPyrr]Cl + NiCl_2_ (3)

From Equation (1), it is suggested that at a pH level exceeding 9, Ni(PrPyrr) remains stable without undergoing hydrolysis, resulting in the presence of a solitary intact metal chelate. Conversely, at a pH level approaching p*K*_1_, a spontaneous hydrolysis process occurs, leading to the demetallation of Ni(PrPyrr) into Ni [(PrPyrr)] and H_2_PrPyrr (the free ligand). Subsequently, at p*K*_2_, [H_2_PrPyrr] undergoes protonation, transforming into [H_3_PrPyrr]^+^ within an acidic medium. This transformation is substantiated by the observations in Figure 2c,d, where Equation (3) is applicable, signifying the instantaneous demetallation of Ni(PrPyrr) at a pH level of 3. The emergence of a UV-vis spectra peak at 330 nm is characteristic of [H_3_PrPyrr]^+^ [47]. Furthermore, as the solution’s pH level is elevated from 3 to 10, a blue shift in the absorption maximum is discerned, shifting from 330 nm to 291 nm. This spectral alteration points to the existence of two distinct species (Figure 2d) and is indicative of a p*K*_1_ value of (6.10 ± 0.03). At this juncture, an equilibrium is established with the free ligand ( [H_3_PrPyrr]^+^ ⇌ H_2_PrPyrr). These UV data have been corroborated with DFT methods (Figures S4 and S5). The pH-dependent speciation of Pd(PrPyrr) has been previously investigated and documented [47].

### 2.3. Assessing the Binding of the Metal Complex Species to HSA

In a previous study [47], we reported the spontaneous hydrolysis behaviour of Pd(PrPyrr) in an aqueous solution, which is pH-dependent and follows a standard two-step p*K*_a_ equilibrium function (H_2_A ⇌ HA + H^+^, *K*_1_; HA ⇌ A + H^+^, *K*_2_). This hydrolysis process results in the formation of three distinct species: PdCl(PrPyrr), [Pd(OH_2_)(HPrPyrr)]Cl, and Pd(OH)(HPrPyrr), all of which are in equilibrium with Pd(PrPyrr). Therefore, at any given pH level there are two species of Pd^II^ complexes present to bind and interact with HSA [47].

**Figure 3:**
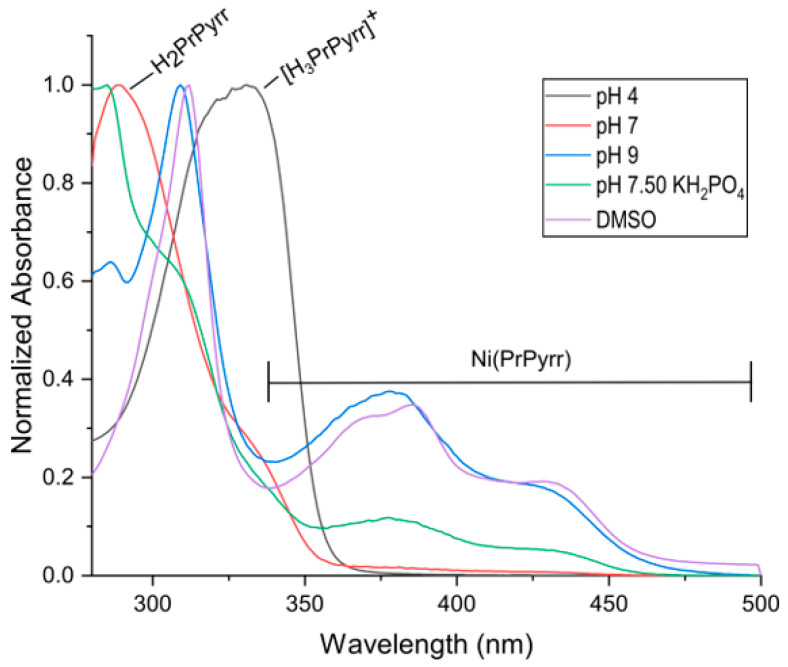
Normalized absorbance spectra of Ni(PrPyrr) bound to HSA measured from 250 to 500 nm in (black) AMT buffer at pH level of 4, (red) AMT buffer at pH level of 7, (blue) AMT buffer at pH level of 10 (AMT buffers were 50 mM Na-acetate, 50 mM MES, and 100 mM Tris-HCl), (green) KH_2_PO_4_ buffer (50 mM, pH level of 7.5); and in (purple) DMSO. The MLCT bands between 375 and 450 nm for Ni(PrPyrr) indicated the chelate was not susceptible to hydrolysis in AMT buffer at pH level of 10, KH_2_PO_4_ buffer, and DMSO, while the chelate rapidly hydrolyzed in AMT buffers at pH levels of 4 and 7.

**Figure 4:**
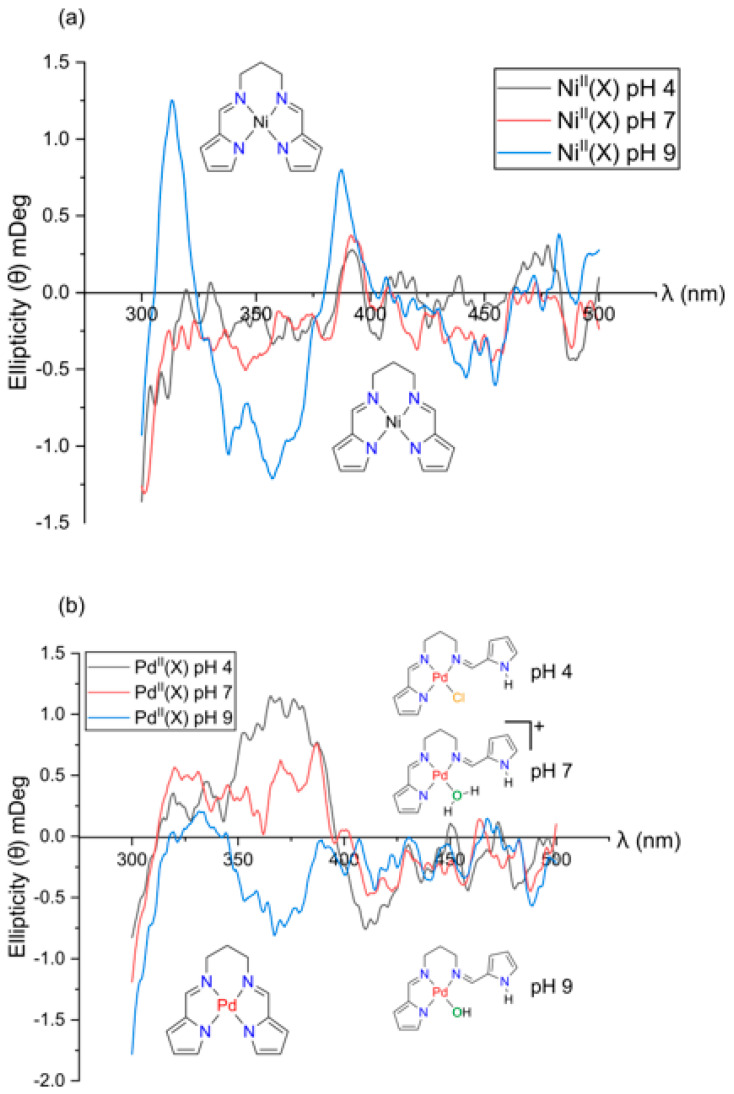
Induced UV–CD (ICD) plots of 15 µM (**a**) Ni(PrPyrr) and (**b**) Pd(PrPyrr) reacted with 15 µM of HSA, where Ni^II^(X) = Ni(PrPyrr) + X and Pd^II^(X) = PdCl(PrPyrr), [Pd(OH_2_)(HPrPyrr)]Cl or Pd(OH)(HPrPyrr). The spectra were recorded at pH levels of 4, 7, and 10 at 310 K (AMT buffers were 50 mM Na-acetate, 50 mM MES, and 100 mM Tris-HCl). The ICD plots allow us to delineate the species of metal complexes present at each pH level. H_2_PrPyrr and [H_3_PrPyrr]^+^ have no ICD signal and they do not meet the threshold of having an absorbance > 350 nm.

From the pH speciation data in Figure 2, the fully intact Ni(PrPyrr) complex is available to bind to HSA at a pH 9 and above. Figure 3 and Figure 4 provide empirical evidence indicating that when Ni(PrPyrr) is introduced into a buffered solution containing native HSA, a portion of the Ni^II^ complex is indeed taken up by the protein. Importantly, the complex is protected from hydrolysis and demetallation under these conditions. Intriguingly, in the AMT buffer at pH levels of 4 and 7, a substantial portion of the Ni^II^ complex undergoes demetallation, resulting in the presence of two distinct species available for binding to HSA. Both species bind to individual sites on HSA, located within a proximity of 15–25 Å from the Trp-214 residue, collectively leading to the quenching of its fluorescence. Specifically, at a pH level of 4, the two species are Ni(PrPyrr) and [H_3_PrPyrr]^+^, whereas at a pH level of 7, they comprise Ni(PrPyrr) and H_2_PrPyrr. These species are not in equilibrium, unlike the Pd(PrPyrr) species. Interestingly, in a study by Sookai et al. [47] it was observed that Ni(PrPyrr) exhibited reduced susceptibility to hydrolysis and demetallation when studied in a KH_2_PO_4_ buffer (50 mM; pH level of 7.5).

### 2.4. Induced Circular Dichroism as a Tool to Investigate the Binding of Small Molecules

In some cases, achiral molecules may exhibit the ability to generate circular dichroism signals, displaying as either left or right-circularly polarized absorptions. This phenomenon can be attributed to the rotational strength of these molecules, which can be described using the Rosenfeld equation (Equation (4)), where *R_j_* is the rotational strength and the scalar product of the vector entities **µ_j_** (electric dipole moment) and $m_{j}^{*}$ (magnetic dipole moment). Both vector transitions (0 → j) are induced by absorption. This describes the rotational strength as the imaginary part of this scalar product using the Rosenfeld equation [48]:

(4) $$R_{j}=I_{m}\;\left({µ}_{j}•\;m_{j}^{*}\right)=I_{m}\;\left(ᴪ\;|µ|{ᴪ}_{j}\right)\;•\left({ᴪ}_{j}\;|m|{ᴪ}_{o}\right)\;$$

From Equation (4), **ᴪ_o_** and **ᴪ_j_** are the wavefunctions of the ground and jth excited states, respectively. From a classical perspective, the scalar product of two vectors produces a non-zero result under the following circumstances: (a) both vectors possess non-zero magnitudes, and (b) they are not perpendicular to each other. As a result, if the electric and magnetic dipole moments satisfy these conditions, the transition will exhibit a rotational strength, leading to the emergence of a CD signal. However, achiral molecules never fulfil both requirements simultaneously [49].

Under specific conditions, however, an achiral molecule can undergo perturbation, leading to a net non-zero rotational strength and the emergence of a CD signal [49]. This phenomenon is termed induced circular dichroism (ICD). Within the scope of this study, we will solely focus on the restriction of conformational freedom due to the interaction of an achiral molecule (guest) with a chiral molecule (host). In this study the host is HSA, and the guests are the divalent metal chelates. From Figure 4 we observe an ICD signal for both Ni^II^(X) and Pd^II^(X), indicating that the most important requirement to observe an ICD signal is met (i.e., the interaction between host and guest induces a specific and mutual orientation in order to observe rotational strengths) [50,51].

The observed ICD signals in Figure 4 are a classic example of a supramolecular complex [51], where HSA is a non-absorbing transparent host and both Ni^II^(X) and Pd^II^(X) are chromophoric guest molecules that bind to and are perturbed by the chiral environment of HSA’s binding site. It is well recognized that HSA has two main small molecule binding sites (Figure 1b) that are mainly hydrophobic [52], namely Sudlow’s Site I (subdomain IIA) and Sudlow’s Site II (subdomain IIIA) [53]. These chiral sites give rise to an induced rotational strength to achiral molecules. It is noteworthy that even though both H_2_PrPyrr and [H_3_PrPyrr]^+^ bind to HSA, their ICD signals are not observed as they do not meet the requirement of the ICD bands being towards the longer wavelengths of HSA and there is overlap. Furthermore, the ICD data in Figure 4 indicate the binding of multiple metal chelates and the chelates bind to HSA intact.

### 2.5. HSA Fluorescence Quenching by Ni^II^(X) and Pd^II^(X)

**Figure 5:**
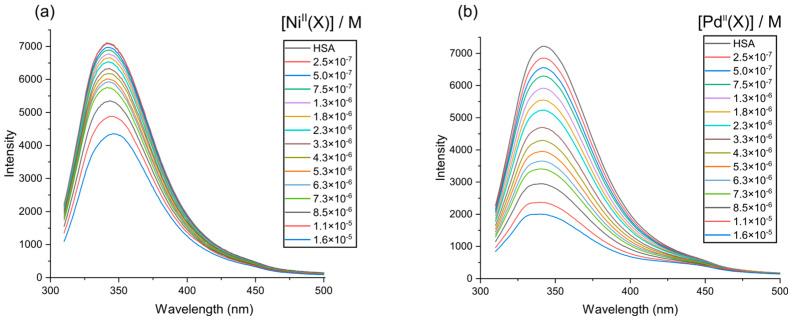
Emission spectra of human serum albumin (HSA, 3.0 μM) recorded as a function of (**a**) Ni^II^(X) and (**b**) Pd^II^(X) at 310 K in AMT buffer (100 mM Na-acetate, 100 mM MES, and 200 mM Tris at a pH level of 7), where Ni^II^(X) = Ni(PrPyrr) + X and Pd^II^(X) = Pd(Cl)(HPrPyrr), [Pd(OH_2_)(HPrPyrr)]Cl or Pd(OH)(HPrPyrr).

When a small molecule binds in close proximity to the aromatic amino acids Trp and/or Tyr within a protein, it induces alterations in the molecule’s intrinsic fluorescence properties [54,55]. HSA has a single Trp residue (Trp-214) embedded in subdomain IIA and is primarily responsible for the intrinsic fluorescence of the protein due to its higher quantum yield and more efficient resonance energy transfer compared to Tyr and Phe [56]. In this study, we aimed to investigate the quenching of the intrinsic emission spectrum of HSA (λ^ex^, 295 nm) by titrating the protein with Ni(PrPyrr) and Pd(PrPyrr) in the concentration range of 0–16 µM with the spectral acquisition measured from 310 to 500 nm (Figure 5a,b) at a pH level of 7, respectively. The $\lambda_{max}^{em}$ was observed at ~343 nm in the constant ionic strength buffer (AMT buffer) at pH levels of 4, 7, and 9, and the intrinsic emission intensity of HSA was quenched monotonically with the addition of each aliquot of the respective divalent metal chelates.

The quenching of HSA fluorescence relies on the overlap between the Trp-214 (donor fluorophore) and the acceptor’s absorbance (i.e., the divalent metal chelates), as well as the spatial proximity of the donor and quencher. Therefore, we can deduce that Ni^II^(X) (where X = Ni(PrPyrr) + [H_3_PrPyrr]^+^ and/or H_2_PrPyrr) and Pd^II^(X) (which refers to PdCl(PrPyrr), [Pd(OH_2_)(HPrPyrr)]Cl, or Pd(OH)(HPrPyrr)), in equilibrium with Pd(PrPyrr), bind to HSA in close proximity of Trp-214 to disorder its microenvironment and quench its fluorescence [57,58]. The mechanisms that may cause fluorescence quenching include ground state complex formation, collisional quenching, energy transfer, or molecular rearrangements [59,60]. At 310 K and a pH level of 7 we found that the binding of Ni(PrPyrr) and H_2_PrPyrr resulted in a redshift $\lambda_{max}^{em}$ of +4 nm, while Pd^II^(X) did not induce a spectral shift. Interestingly, at 310 K and at a pH level of 4 Ni(PrPyrr) and [H_3_PrPyrr]^+^, and at a pH level of 9, Ni(PrPyrr) induced red and blue shifts $\lambda_{max}^{em}$ of +10 and −10 nm, respectively, while Pd^II^(X) at a pH level of 4 did not induce a spectral shift, but, at a pH level of 9 a blue shift $\lambda_{max}^{em}$ of +8 nm was observed.

The $\lambda_{max}^{em}$ shift data suggest that the microenvironment surrounding Trp-214 in HSA has become more hydrophilic at pH levels of 4 and 7, while at a pH level of 9 it becomes more hydrophobic [61]. Two potential explanations accounting for the $\lambda_{max}^{em}$ red shifts are as follows: (i) the divalent Schiff base chelates bind to HSA sufficiently close to Trp-214 to induce direct electronic polarization of the indole ring in the residue, and/or (ii) the disruption of the ordered water molecules located within 15–25 Å of Trp-214 can lead to an orientation-dependent polarization of the fluorophore. It is possible that both explanations result in the lowest energy ^1^A_1_ → ^1^L_a_ ground state transition of Trp’s indole ring [57], thereby causing the $\lambda_{max}^{em}$ shift. Pd^II^(X) had no $\lambda_{max}^{em}$ shift at pH levels of 4 and 7, suggesting that it did not induce sufficient direct electronic polarization of the indole ring in the tryptophan residue to cause a shift in the HSA emission spectrum. However, we previously reported Ni(PrPyrr) and Pd(PrPyrr) binding to the N-form of HSA (50 mM KH_2_PO_4_; pH level of 7.5) resulting in $\lambda_{max}^{em}$ red shift of 0.91 nm and $\lambda_{max}^{em}$ blue shift of 5.6 nm, respectively [47].

To understand the vastly dissimilar $\lambda_{max}^{em}$ shifts in this study, we must consider the changes to not only the binding of the metal Schiff base chelates to HSA, but also the change in HSA’s environment as well. Subdomains IIA and IIIA are stabilized by hydrophobic interactions and salt bridge interactions [62,63]. At a pH level of 4, HSA is dominantly in the F-form (and only transitions into the E-form below a pH level of 3) [64]. There is a negligible difference in the secondary structure of HSA between F and N-forms. This may account for the similar $\lambda_{max}^{em}$ shifts observed at pH levels of 4 and 7. At a pH level of 9, most of HSA is in the B-form [65] and upon the binding of the metal chelates a $\lambda_{max}^{em}$ blue shift is observed. Unlike the N-form of HSA, the B-form is only partially characterized. In particular, the N to B-form transition has been characterized by ^1^H NMR and it was proposed that the heart shaped N-form undergoes conformational changes to a more open ellipsoid conformation [66]. Furthermore, HSA would carry a net negative charge; therefore, it is unlikely that the $\lambda_{max}^{em}$ shifts in Figure S2 are solely attributed to metal Schiff base chelates binding sufficiently close to HSA’s Trp-214. But the $\lambda_{max}^{em}$ shifts phenomena are also a result of the buffer’s ionic strength and pH-dependent conformational changes of albumin.

### 2.6. Fluorescence Quenching Mechanism

The quenching mechanism of a protein’s intrinsic fluorescence can be categorized as static, dynamic, or mixed quenching, and is typically analyzed using the Stern–Volmer equation (Equation (5)) [55]:

(5) $$I_{0}/I=1+K_{SV}\left[Q\right]=1+k_{q}\tau_{0}\left[Q\right]$$

where *I_o_* refers to the fluorescence intensity of HSA in the absence of a quencher (Ni^II^(X) or Pd^II^(X) chelates herein) and *I* refers to the fluorescence intensity of HSA in the presence of the quencher. K*_SV_* is the Stern–Volmer constant (M^−1^) and [*Q*] is the molar concentration of the quencher, k*_q_* is the bimolecular quenching rate constant (M^−1^ s^−1^), and *τ*_0_ is the average lifetime of HSA fluorescence in the absence of any quencher (5.28 ± 0.03 ns [67], 5.60 ± 0.10 ns [68], 6.72 ± 0.07 ns [56]; mean = 5.9 ± 0.76 ns). Typically, the quenching mechanism can be distinguished by analyzing the HSA•{ligand} complex’s fluorescence as a function of viscosity and temperature [69].

**Figure 6:**
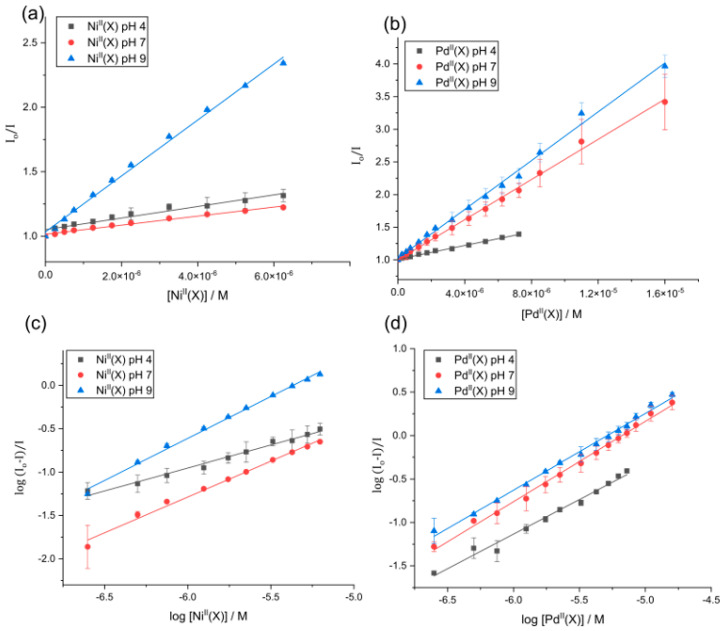
Stern–Volmer plot for HSA (3 µM) in AMT buffer (100 mM Na-acetate, 100 mM MES, and 200 mM Tris at pH levels of 4, 7, and 9) recorded as a function of (**a**) Ni^II^(X) (R^2^ = 0.998, 0.988, and 0.950) and (**b**) Pd^II^(X) (R^2^ = 0.997, 0.997, and 0.995) vs. fluorescence intensity ratio (*Io/I*) at 310 K, where Ni^II^(X) = Ni(PrPyrr) + X and Pd^II^(X) = PdCl(PrPyrr), [Pd(OH_2_)(HPrPyrr)]Cl, or Pd(OH)(HPrPyrr). Each plot represents the average of three independent experiments and the error bars represent ESDs. The data are well fitted to linear Equation (1), suggesting a single quenching mechanism is dominant. Double logarithm plot of the fractional change in fluorescence intensity for human serum albumin (HSA, 3.0 μM) recorded as a function of the concentration of (**c**) Ni^II^(X) (R^2^ = 0.984, 0.991, and 0.996) and (**d**) Pd^II^(X) (R^2^ = 0.984, 0.995, and 0.994) (constant ionic strength AMT buffer at pH levels of 4, 7, and 9). Error bars are ESD’s based on the average of three independent determinations. The data are described by Equation (3), which affords the affinity constant and stoichiometric coefficient for the reaction (Table 1).

The Stern–Volmer plots for the fluorescence emission quenching of HSA as a function of temperature (310 K) and [Ligands] (the term “Ligands” is used in a biochemical context; for the complexes Ni^II^(X) or Pd^II^(X) chelates = the Ligands or quenchers herein) are presented in Figure 6a,b. From the slope of the least squares fit of Equation (5) to the data, the bimolecular fluorescence quenching rate constant (k*_q_*) for the HSA-Ligand interaction can be calculated (Equation (6)):

(6) $$\;k_{q}=K_{SV}/\tau_{0}$$

The K*_SV_* and k*_q_* values describing the interaction of the Ligands and HSA are summarized in Table S1. Typically a linear Stern–Volmer plot indicates that a single quenching mechanism is dominant, i.e., either static (binding-related) or dynamic (diffusion-limited collisional) [54,55].

The quenching mechanism can be identified by the K*_SV_* dependence on temperature. If K*_SV_* values increase with increasing temperature, the dominant mechanism is dynamic because there is an increase in the HSA•{Ligands} formation constant. However, if the reverse is observed, the quenching mechanism is static, since there is a decrease in the HSA•{Ligands} formation constant [70].

The Stern–Volmer plots for the Ligands binding to HSA were linear, indicating that a single quenching mechanism is dominant—either static (binding-related) or dynamic (diffusion-limited collisional) [54,55]. The K*_SV_* values of both Ni^II^(X) and Pd^II^(X) complexes decreased with increasing temperature, consistent with a static quenching mechanism. The k*_q_* values for the Ligands all exceed the diffusion-controlled limit (1 × 10^10^ M^−1^ s^−1^) [71] by 2 to 3 orders of magnitude, which is consistent with significant HSA–ligand binding interactions rather than non-specific diffusion-controlled collisional interactions [59]. The K*_SV_* values were largest at a pH level of 9 and decreased with decreasing pH levels; however, a strong ligand-binding phenomenon was observed throughout the pH range. The K*_SV_* values are consistent with those reported of the same molecules in the KH_2_PO_4_ buffer [47]. A summary of the K*_SV_* and k*_q_* values obtained for the interaction of the Ligands with HSA is reported in Table S1.

### 2.7. Ligand Binding Equilibrium Constants

The quenching of intrinsic HSA fluorescence emission as a function of [Ligand] can be used to calculate the biophysical binding parameters for the interaction of the divalent metal chelates with HSA, i.e., the affinity constant (K*_a_*) and the reaction stoichiometry (n). Both factors can be obtained from a double log plot and least squares fit of the emission quenching data as a function of the increasing Ligand concentration (Equation (7)) [53],

(7) $$\log\left(\frac{I_{0}-I}{I}\right)={\mathrm{logK}}_{a}+\mathrm{nlog}\left[Q\right]$$

where the intercept and the gradient of the slope equate to log K*_a_* and n, respectively. The data for the Ligands at different pH levels and temperatures are summarized in Table 1 and are plotted in Figure 6c,d.

The affinity constant (K*_a_*) for both Ni^II^(X) and Pd^II^(X) decreases with increasing temperature, consistent with a static quenching mechanism [53,72]; Table 1 shows the log K*_a_* values of Pd^II^(X) > Ni^II^(X) at pH levels of 4 and 7. This is not surprising, as we have previously observed that the metal chelates possess a higher binding affinity for HSA as compared to the free ligand, and, Ni^II^(X) was observed to demetallate in the AMT buffer at pH levels of 4 and 7 [47]. The values of the log K*_a_* for the Pd^II^ chelates range from 3.71 to 5.51 at a pH level of 4, and 4.86 to 5.57 at a pH level of 7 (288–310 K), compared to the Ni^II^(X) ligands that ranged from 2.18 to 4.62 at a pH level of 4 and 3.81 to 4.43 at a pH level 7 (288–310 K). The log K*_a_* values of Pd^II^(X) and Ni^II^(X) ligands are akin to the product equilibrium constant *β*_2_, where $log\beta_{2}=logK_{1}+logK_{2}$, accounting for why the affinity constant is an order of magnitude larger than the K*_a_* values measured for the Ni^II^(X).

At a pH level of 9, the data are vastly different, which shows the log K*_a_* for Ni(PrPyrr) > Pd^II^(X). From Figure 2 we observe that at an alkaline pH level, only Ni(PrPyrr) is present, while two Pd^II^(X) species are present. The Ni(PrPyrr) chelate has a significantly higher log K*_a_* at a pH level of 9 when compared to the Pd^II^(X) chelates. However, at pH levels of 4 and 7, due to significant demetallation of the Ni(PrPyrr), the Pd^II^(X) chelates had the highest binding constants. Additionally, two Pd^II^ species are present at all pH levels and bind with HSA concurrently.

For Pd^II^(X), the log K*_a_* values are a product of the product equilibrium constant $\beta_{2}$, where $log\beta_{2}=logK_{1}+logK_{2}$. It is plausible that at a pH level of 9, two Ni(PrPyrr) complexes bind to HSA; mass spectroscopy data have shown this hypothesis to be plausible [47]. In all cases, we suspect multiple binding, $K_{1}$ and $K_{2}$; for the reaction of HSA with the Ni(PrPyrr) (pH level of 9) and Pd^II^(X) (all pH levels), systems are unresolvable, reflecting concurrent ligand binding.

### 2.8. Thermodynamics of the Ligands Binding by HSA

**Figure 7:**
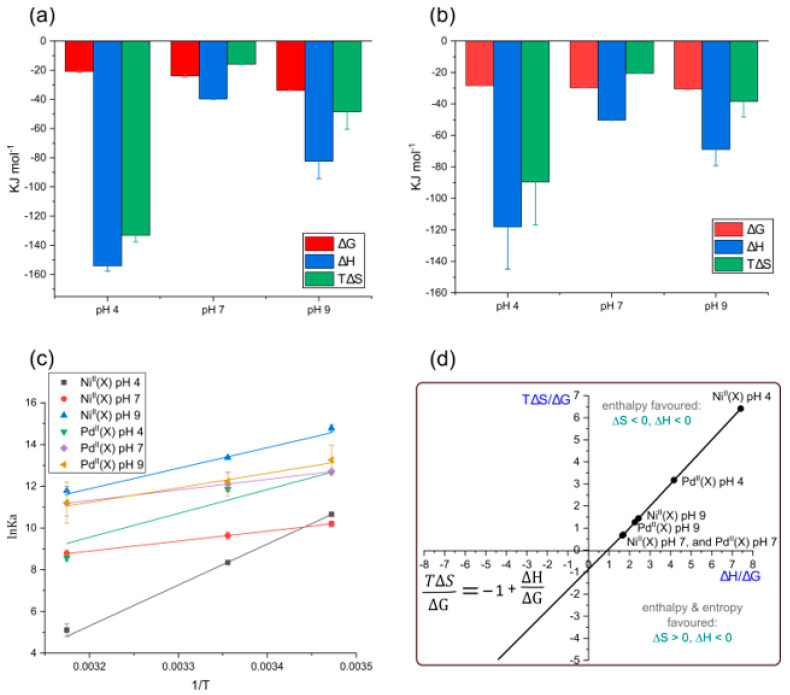
Thermodynamic parameters governing the reactions of (**a**) Ni^II^(X) (**b**) Pd^II^(X) with HSA (*T* = 298 K), where Ni^II^(X) = Ni(PrPyrr) + X. Pd^II^(X) = Pd(Cl)(PrPyrr), [Pd(OH_2_)(HPrPyrr)]Cl, or Pd(OH)(HPrPyrr). (**c**) Linear van’t Hoff plots (Equation (8)) for the reactions of the ligands with HSA in constant ionic strength AMT buffer at pH levels of 4, 7, and 9. Error bars are derived from averaging triplicate measurements. Pearson’s *r*-values for Ni^II^(X) fits were 0.984, 0.999, 0.962, at pH levels of 4, 7, and 9, respectively. Pearson’s *r*-values for Pd^II^(X) fits were 0.989, 0.999, 0.999, at pH levels of 4, 7, and 9, respectively. (**d**) Plot of the Gibbs–Helmholtz relationship for the reaction of Ni^II^(X) and Pd^II^(X) with HSA at 298 K in constant ionic strength AMT buffer at pH levels of 4, 7, and 9. The straight line fit of the data gives *R*^2^ = 0.999 For all reactions, ∆*G <* 0. The plot highlights how the identity of the d^8^ metal ion influences the reaction thermodynamics.

The reactions of the ligands follow a linear van’t Hoff relationship; plots for triplicate measurements are presented in Figure 7a. Under non-standard conditions, the thermodynamic parameters, i.e., enthalpy change (*∆H*), entropy change (*∆S*), and Gibbs free energy change (*∆G*), may be deduced from Equations (8) and (9).

(8) $$lnK_{a}=-\frac{\Delta H}{RT}+\frac{\Delta S}{R}$$

(9) ΔG=H –TΔS 

Table 2 summarizes the thermodynamic parameters for the reaction of the current ligands with HSA in the constant ionic strength AMT buffer. All three reactions are exergonic [53], with *∆G* values of −20.8, −23.9, and −33.8 kJ mol^−1^ for Ni(PrPyrr) + X at pH levels of 4, 7, and 9, respectively, at 298 K. Pd^II^(X) *∆G* values were −28.3, −29.7, and −30.5 kJ mol^−1^ at pH levels of 4, 7, and 9, respectively, at 298 K.

The enthalpy values (*∆H*) for reactions of Ni^II^(X) and Pd^II^(X) with HSA are exothermic and are summarized in Table 2. Figure 7b,c highlights the dominance of the enthalpy term for the reactions of each divalent metal chelates with HSA. It is well recognized that HSA has two main small molecule binding sites that are mainly hydrophobic [52], namely Sudlow’s Site I (subdomain IIA) [53] and Sudlow’s Site II (subdomain IIIA) [53] (Figure 1b). The large negative *T∆S* values for each divalent metal chelate binding recorded here imply enhanced conformational rigidity and minimal disruption of the HSA’s backbone structure or ordered water molecules and further commensurate the metal chelate uptake in one or more hydrophobic pockets. London dispersion forces (LDF) are likely the dominant binding forces, which is a deduction confirmed by our molecular docking and QM:MM simulations (vide infra). When HSA is charged (i.e., positive (pH level of 4) or negative (pH level of 9)) the *∆H* and *T∆S* terms are the more negative when compared to a neutral pH level. This is expected, as the amino acid residues at pH levels of 4 and 9 are now charged and the HSA–ligand system is now more energetically favored for non-covalent interactions [73]. The *∆G* values became increasingly negative at more alkaline pH levels.

In Figure 7d we used the Gibbs–Helmholtz relationship (Equation (8)) and the experimental thermodynamic data (Table 2) to determine the influence that the ligands have on the thermodynamic parameters for the complex formation with HSA. From Figure 7d, when *T∆S/∆G* is plotted against *∆H/∆G*, the resulting fit is linear with *x*- and *y*-intercepts of exactly 1. Reactions for the ligands at all three pH levels with HSA are enthalpically driven *(∆H* < 0) and are based in the top right quadrant. In this quadrant, spontaneity is assured as changes in *∆H/∆G* are compensated for by commensurate changes in *T∆S/∆G*. Importantly, under all conditions assessed, *T∆S/∆G* and *∆H/∆G* decrease together, giving the order Pd^II^(X) < Ni^II^(X) + free ligand which follows the principal quantum number for the metal ion (4d *<* 3d). This trend indicates that the thermodynamics of the metal Schiff base chelates binding to HSA depends directly on increasing the electron density present in the complexes (i.e., LDF). The results mirror the trend previously reported by Sookai et al. [47].

### 2.9. Ligand and pH-Induced HSA Secondary and Tertiary Structure Changes

**Figure 8:**
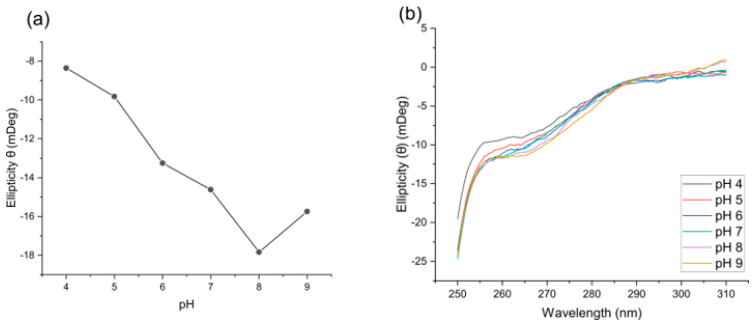
(**a**) Normalized far–UV CD of native HSA (500 nM) monitoring the α-helix n–π* transition at 222 nm from pH levels of 4 to 9 in AMT (100 Na-acetate mM, 100 mM MES, and 200 mM Tris buffer) at 310 K. (**b**) Near-UV CD data of native HSA (15 μM) monitoring the tertiary structure of HSA in AMT buffer from pH levels of 4 to 9.

In Figure 8a we evaluated the change in HSA’s secondary structure using far UV–CD spectroscopy at various pH levels and a constant ionic strength [31,32]. HSA is an α-helix rich protein, which was confirmed by the characteristic double minima at 208 nm and 222 nm (Figure S3) that are associated with the π-π* and n–π* excitations, respectively [74]. The transitions originate from the amide bonds of the peptide backbone [31,32].

The varying pH levels altered HSA’s secondary structure. This was confirmed by the change in ellipticity of HSA in the far UV region (Figure 8a). The highest ellipticity was observed at a pH level of 8 when HSA is dominantly in the N-form. As HSA transitions from the N-form to the B-form (pH level of 9) the ellipticity (i.e., the protein’s secondary structure conformational stability) of the protein decreases, likely due to the “heart-shape” becoming less ordered and more elongated [75]. When HSA was in the F-form (pH levels of 4–6) it possessed the lowest ellipticity, indicating that the protein’s secondary structure was most perturbed and partially unfolded (i.e., the most elongated form) [34]. It should be noted that the extreme forms of HSA were not analyzed in this study, i.e., the E-form (pH level < 2.7) and the A-form (pH level > 10). It is unlikely HSA will be exposed to such extreme pH levels in vivo.

Near UV-CD spectroscopy is a powerful technique for gaining insight into a protein’s tertiary structure. This technique probes the electronic structure of Trp (285–300 nm), Tyr (275–285 nm), and Phe (250–270 nm), enabling the examination of conformational changes within the protein’s tertiary structure. For native HSA, the ellipticity of the protein increased with increasing pH levels (Figure 8b). This phenomenon may be attributed to distinct structural elements analyzed with both techniques. Far UV-CD focuses on the protein’s secondary structure (i.e., α-helices, β-sheets, and random coils), while near UV-CD focuses on a protein tertiary structure and CD active chromophores (i.e., disulfide bonds and aromatic amino acid residues) [74,76].

**Figure 9:**
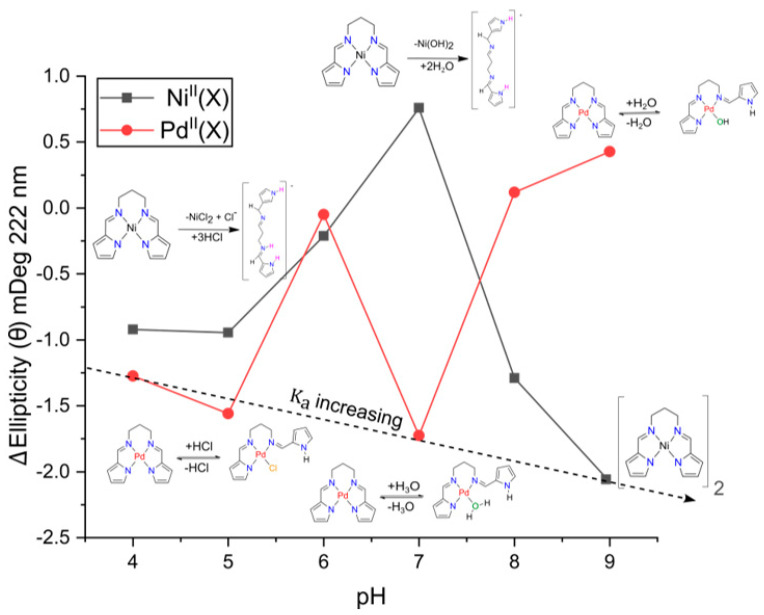
Plots of the change in far UV–CD spectra of HSA•{ligand} compared to native HSA. The change in ellipticity monitored the α-helix n–π* transition at 222 nm from pH levels of 4 of 9 in AMT buffer (100 mM Na-acetate, 100 mM MES, and 200 mM Tris buffer) at 310 K. At each pH level there is 5 µM of ligand present. Trend line passing through minima points at pH levels of 4, 7, and 9 indicates increasing binding affinity of ligands to HSA.

As shown in Figure 9, we measured the alteration in HSA’s secondary structure upon binding of the ligands by monitoring the change in the n–π* transition at 222 nm from pH levels of 4 to 9. This was achieved by measuring the difference in ellipticity of native HSA at a particular pH level and HSA•{ligand} under the same conditions.

Ni(PrPyrr) was prone to hydrolysis at pH levels of 4 to 8, while at a pH level of 9, Ni(PrPyrr) was present to react with HSA (Figure 2). At pH levels of 4 and 5, Ni^II^(X) induced a conformational alteration in the F-form of HSA’s secondary structure, resulting in the n–π* transition to decrease by ~1 mDeg. At pH levels of 6 and 7, the F-form of HSA was transitioning to the B-form, and at a pH level of 6, minimal perturbations in HSA’s secondary structure were observed (~0.2 mDeg); however, at a pH level of 7, the n–π* transition increased by ~1 mDeg. At pH levels of 8 and 9, Ni(PrPyrr) was the dominant species present and caused the greatest conformational change in HSA’s secondary structure.

At first glance, there is no observable trend of the Pd^II^(X) species. However, this is due to the different species existing at each pH level. At pH levels of 4 and 5 (Pd(PrPyrr) + HCl ⇌ Pd(Cl)(HPrPyrr)) upon the binding of both species to HSA, there is a decrease in HSA’s secondary structure. Similarly, at a pH level of 7 (Pd(PrPyrr) + H_2_O + HCl ⇌ [Pd(H_2_O)(HPrPyrr)]Cl) had the same effect. The Pd^II^(X) chelates decrease the ellipticity of the protein by decreasing the n–π* transition and reducing HSA’s conformational stability. At alkaline pH levels (Pd(PrPyrr) + H_2_O ⇌ Pd(OH)(HPrPyrr)) had minimal influence in altering the secondary structure of HSA. Unsurprisingly, we found a correlation between the *∆*mDeg and log K*_a_*. The stronger binding ligands perturbed HSA more significantly and resulted in a greater change in *∆*mDeg. This is in agreement with data reported previously by Sookai et al. [47].

### 2.10. In Silico Binding Site Determination

**Figure 10:**
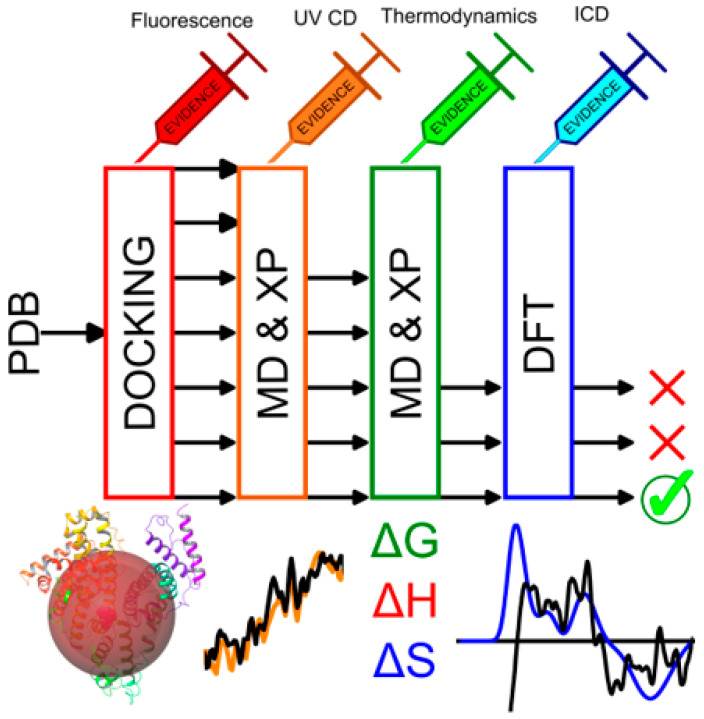
Injecting experimental evidence into in silico workflows using fluorescence and CD spectroscopy. Unknown ligand binding site determination on blood transport protein, HSA.

The determination of unknown ligand binding sites is a challenge without X-ray crystal data. Here, we present an in silico workflow that utilizes only one protein structure from the PDB and incorporates experimental fluorescence and CD data to determine the likely ligand binding sites on HSA. The workflow is presented in Figure 10 below. The process begins by selecting a PDB crystal structure with good resolution and minimal disorder.

An initial docking run is performed with constraints based on fluorescent data; if the Stern–Volmer plots show linearity over the full concentration range, it can be assumed that ligand binding is taking place within 15–20 Å of the major fluorophore, but up to 25 Å is also possible. HSA is an ideal protein for fluorescent measurements because it only has a single tryptophan residue which acts as the main probe. This has been shown as red spheres on a cartoon model of HSA; the spheres radiate from Trp-214 to a distance of between 15–20 Å. Hence, due to the trends in fluorescent data, ligand binding must take place within this radius and any ligands docked beyond this must be eliminated as potential binding sites. Ligands docked within an appropriate distance from Trp-214 are then subjected to molecular dynamics [77] (MD) equilibration and subsequent extra precision (XP) docking [78].

The MD trajectory will eliminate any poorly docked ligands and well docked ligands will equilibrate over 100 ns such that the protein conformation around the ligand has reached an average conformation. Once a stable protein–ligand complex has equilibrated, the ligand is removed, and XP docking is performed again. This may either improve the G-score of the docked ligand in the same site, or, due to the static nature of docking, after MD equilibration, new binding sites and ligand poses may be generated. The experimental evidence must be considered at every stage of docking. Hence, if near UV-CD data indicate that the Cys-Cys chromophore is perturbed upon ligand binding, then ligands docked >10 Å away from this moiety should be eliminated. The near UV-CD data may also be used to constrain the docking. For example, if the Tyr chromophore is perturbed upon ligand binding, then positional constraints may be employed to restrict ligand binding to within 10 Å of a selected residue.

Additionally, the thermodynamic data must be considered. With a ΔS < 0, this suggests ligand binding is causing conformational changes in the protein and that the ligand is buried within a cavity of HSA. This also suggests that disordered water within a hydrophobic pocket of the blood transport protein is being displaced. Hence, any surface-bound or largely polar binding sites must be eliminated from the docking workflow. Because G-scores are unreliable [79], they are not considered when determining a potential binding site; only the empirical data are used to discriminate ligand binding sites. The G-score is only considered when selecting a ligand pose within a particular protein site. Once a final MD equilibration is performed, and docked ligands corroborate the experimental evidence, the protein–ligand complexes that satisfy these experimental restrictions are subjected to time-dependent-DFT (TD-DFT) simulations. The macromolecular system is subjected to multilayer ONIOM [80] calculations where the ligands and selected residues are modelled in a quantum layer using TD-DFT theory. The protein itself is modelled in a mechanics layer using force field theory. The output of these calculations produces a CD spectrum that can be correlated to the experimental ICD spectrum. The protein–ligand complex that best corroborates all the empirical data is determined to be the system observed experimentally, using fluorescence and CD spectroscopy.

The workflow presented in Figure 10 was used to determine the ligand binding sites for Ni^II^(X) and Pd^II^(X) at pH levels of 4, 7, and 9. The results for Ni(PrPyrr) at a pH level of 9 are presented in Figure 11 and Figure 12 below. The results for Ni^II^(X) and Pd^II^(X) at all pH levels are presented in the Supporting Information (Figures S6–S16).

**Figure 13:**
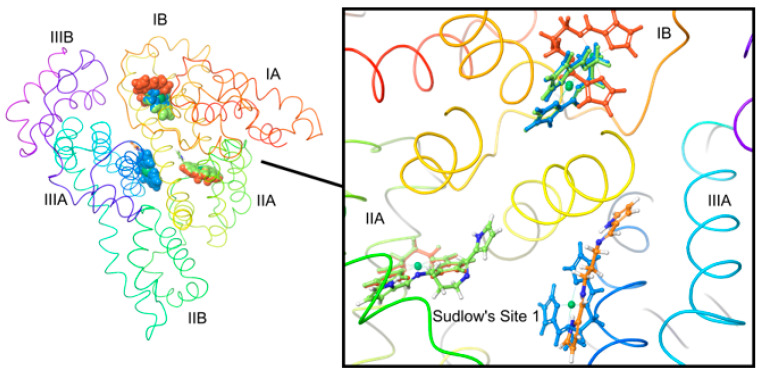
HSA docked with Ni^II^(X) at pH levels of 4, 7, and 9. Ni(PrPyrr) docked at a pH level of 4 shown in orange, pH level of 7 shown in green, and pH level of 9 shown in blue; the Ni^II^ ion is shown as metallic-green. The ligand H_2_PrPyrr docked at a pH level of 7 is shown in green with nitrogens as blue and hydrogens as white. The ligand [H_3_PrPyrr]^+^ docked at a pH level of 4 is shown in orange with nitrogens blue and hydrogens as white. At a pH level of 4, there is significant demetallation and a species distribution exists where both HSA•{ [H_3_PrPyrr]^+^}•{Ni(PrPyrr)} and HSA•{Ni(PrPyrr)} are present in solution. When [H_3_PrPyrr]^+^ is bound at Site 1, Ni(PrPyrr) is directed to subdomain IB. However, a population exists where Ni(PrPyrr) is bound at Site 1 at a pH level of 4. This is also observed at a pH level of 7 when demetallation takes place in the AMT buffer used. Hence, at a pH level of 7, the species distribution HSA•{H_2_PrPyrr}•{Ni(PrPyrr)} HSA•{Ni(PrPyrr)} is present. At a pH level of 9, Ni(PrPyrr) is stable and binds has with 2 equivalents, resulting in the highly favorable binding affinity measured. The species present at a pH level of 9 is thus HSA•{Ni(PrPyrr)}_2_ where one chelate is bound at Site 1 and the other at subdomain IB.

At a pH level of 9, Ni(PrPyrr) is stable in the AMT buffer and binds HSA with two equivalents of metal complex resulting in the highest observed log K_a_. Ni(PrPyrr) binds to HSA at a pH level of 9 at Sudlow’s Site 1 within 3 Å of Trp-214 and at subdomain IB so that the solution species is HSA•{Ni(PrPyrr)}_2_. Each metal chelate is stabilized by numerous hydrophobic interactions to residues such as tyrosine, phenylalanine, and isoleucine. At a pH level of 7, Ni(PrPyrr) undergoes irreversible hydrolysis to produce H_2_PrPyrr in significant quantities. As a result, the ligand H_2_PrPyrr is present and available to bind to HSA at Site 1, leaving the site unavailable for binding Ni(PrPyrr). The metal complex is thus directed to subdomain IB when H_2_PrPyrr is bound to Site 1 resulting in the species HSA•{H_2_PrPyrr}_Site 1_•{Ni(PrPyrr)}_IB_. However, a small portion of metal chelate may bind at Sudlow’s Site 1 where it is protected from hydrolysis, resulting in the species HSA•{Ni(PrPyrr)}_Site 1_. Hence, at a pH level of 7, a species distribution exists where both chelate-bound proteins are present in the solution. This is also observed for Ni^II^(X) at a pH level of 4, but the ligand is protonated to form [H_3_PrPyrr]^+^. The HSA ligand binding sites for Ni^II^(X) at all pH levels have been presented below in Figure 13.

The Pd^II^(X) species were stable in AMT buffer at all pH levels and displayed reversible hydrolysis. The products of the hydrolysis of Pd(PrPyrr) are pH dependent, where the substituted ligand is chloro, aqua, or hydroxyl at pH levels of 4, 7, or 9, respectively. The Pd(PrPyrr) chelate binds at Sudlow’s Site 1 within 7 Å of Trp-214 at all pH levels. The chelate stability can be attributed mostly to London dispersion forces between the ligand and hydrophobic residues within the pocket surrounding Trp-214.

The products of hydrolysis, Pd(Cl)(HPrPyrr), [Pd(H_2_O)(HPrPyrr)]^+^, and Pd(OH)(PrPyrr), were all found to bind to HSA at subdomain IIB at the relevant pH level. Subdomain IIB is rich with aromatic residues phenylaniline and tyrosine, and these chromophores are significantly perturbed in the near UV-CD region upon chelate binding. These hydrophobic sites, Sudlow’s Site 1 and subdomain IIB, stabilize the chelate through dispersion forces which result in a favorable change in enthalpy. The displaced disordered waters and restricted protein conformation upon chelate binding result in an unfavorable change in entropy. However, the enthalpic contribution of Ni^II^(X) and Pd^II^(X) binding to HSA at all pH levels results in a net favorable change in Gibbs free energy that increases with increasing pH levels. Although in silico and empirical data are well corroborated, only the X-ray crystal or Cryo-EM data will be definitive.

## 3. Materials and Methods

### 3.1. Experimental Methods

All solvents (HPLC grade) and chemical synthons were used as received from Merck Sigma-Aldrich^®^ without further purification. Human serum albumin (HSA) was purchased from Sigma and used as received without further purification. Ultrapure water (Type I) was produced using a Merk-Millipore Direct-Q^®^ 3 UV Water Purification System.

### 3.2. Instrument Basic Methods

Proton and carbon NMR spectra were recorded on Bruker Avance III 400 NMR spectrometers at ^1^H frequencies of 400 MHz, and ^13^C frequencies of 100 MHz. Spectra were recorded at 300 K with 5 mm BBOZ or TBIZ probes. Chemical shifts for both proton and carbon were referenced using the solvent signal. MestReNova (version 14.2.1-27684) was used to analyze NMR spectra. FT-IR spectra of powder samples were recorded using a Bruker Alpha FTIR spectrometer incorporating a Bruker Platinum^®^ diamond ATR sampling accessory. Spectra were analyzed using the OPUS software package on the spectrometer (version 7.5). Mass spectra were recorded with a Bruker Compact Q-TOF high-resolution mass spectrometer using Bruker Daltronics HyStar 3.2 SR4 software. Bruker Compass Data Analysis software (version 4.3) was used to analyze chromatograms. Samples of pure compounds (typically ca. 10 μg/mL) were prepared in HPLC-grade acetonitrile or ethanol for metal chelates and HPLC-grade methanol for ligands. Solutions were acidified using 0.1% (*v/v*) formic acid to obtain spectra in ESI+ mode. Electronic spectra were recorded using either a PerkinElmer Lambda 365 double-beam spectrometer connected to a Peltier controller or a multicell thermostatic cell block. The spectral data were analyzed with the spectrometer software or Origin Pro 2022. Spectra were recorded (10-mm pathlength quartz cuvettes) as a function of concentration for both characterization and the determination of molar absorptivity constants.

### 3.3. Compound Synthesis

*N*,*N*′-bis [(1*E*)-1*H*-pyrrol-2-ylmethylene]propane-1,3-diamine (H_2_PrPyrr). The synthetic method was adapted from the method described by Munro et al. [82]. Briefly, pyrrole-2-carboxaldehyde (0.5231 g, 7.5 mmol) and 1,3-diaminopropane (0.275 g, 3.37 mmol) were refluxed in ethanol (15 mL) for 120 min. During refluxing, the reaction mixture changed from pale yellow to a clear straw yellow. Ethanol was then removed by rotary evaporation, resulting in a viscous, orange-colored oil. Finally, the viscous orange oil was dissolved in 2 mL of dichloromethane and layered with hexane in a round bottom flask. The solution was left overnight, affording a faint yellow powdered product. H_2_PrPyrr (1.2188 g, 80% yield) was analyzed by ^1^H NMR, ^13^C NMR, FT-IR, and UV-visible spectroscopy, producing data in agreement with the earlier literature [82]. ^1^H NMR (400 MHz, chloroform-*d*, 300 K) [δ, ppm]: 1.98 (t, *J* = 6.8 Hz, 2H, H-7), 3.61 (td, *J* = 6.8, 1.2 Hz, 4H, H-6), 6.23 (dd, *J* = 3.6, 2.6 Hz, 2H, H-2), 6.48 (dd, *J* = 3.6, 1.4 Hz, 2H, H-3), 6.88 (t, *J* = 1.9 Hz, 2H, H-1), 8.03 (s, 2H, H-5). ^13^C NMR (101 MHz, chloroform-*d*, 300 K) [δ, ppm]: 32.29 (C-7), 57.80 (C-6), 109.59 (C-2), 114.13 (C-3), 122.04 (C-1), 129.86 (C-4), 151.85 (C-5). IR (KBr pellet, cm^−1^): 3112w δ(NH, pyrrole), 3053m br *v*(CH, imine), 2941m *v*(CH, CH_2_CH_2_CH_2_), 2847 *v*(CH, CH_2_-N=CH), 1634s br *v*(C=N). UV-vis (ethanol) [λ_max_/nm, ε/mol^−1^ dm^3^ cm^−1^]: 289, 3.20 × 10^4^.

2,2′-{propane-1,3-diylbis [azanylylidene(*E*)methanylylidene]}di(pyrrol-1-ide)nickel(II), Ni(PrPyrr). The complex was synthesized by modification of methods in the literature [22,47]. ^1^H NMR (400 MHz, DMSO-*d*6, 300 K) [δ, ppm]: δ 7.57 (s, 2H), 6.80 (s, 2H), 6.50 (d, *J* = 3.5 Hz, 2H), 5.97 (dd, *J* = 3.6, 1.9 Hz, 2H), 3.23–3.08 (m, 4H), 1.70 (p, *J* = 4.3, 3.9 Hz, 2H). ^13^C NMR (101 MHz, DMSO-*d*6, 300 K) [δ, ppm]: δ 162.47, 140.20, 136.17, 116.37, 112.00, 52.41, 28.99. FT-IR (powder, cm^−1^): 3092 m br *v*(CH, imine), 3074 m *v*(CH, CH_2_C*H*_2_CH_2_), 2936m *v*(CH, C*H*_2_-N=CH), 1579s br *v*(C=N). UV-vis (acetonitrile) [λ_max_/nm, ε/mol^−1^ dm^3^ cm^−1^]: 313, 2.68 × 10^4^; 374, 8.56 × 10^3^; 385, 9.16 × 10^3^; 432, 4.92 × 10^3^. HRMS (*m*/*z*): [M + H]^+^ calcd. for C_13_H_14_N_4_Ni, 285.0645; found, 285.0688.

2,2’-{propane-1,3-diylbis [azanylylidene(*E*)methanylylidene]}di(pyrrol-1-ide)palladium(II), Pd(PrPyrr). The complex was synthesized by the method in the literature. [47] ^1^H NMR (400 MHz, chloroform-*d*, 300 K) [δ, ppm]: δ 7.52 (s, 2H), 7.16 (s, 2H), 6.70 (d, *J* = 3.7 Hz, 2H), 6.22 (dt, *J* = 3.5, 1.4 Hz, 2H), 3.52–3.39 (m, 4H), 1.98 (p, *J* = 4.7 Hz, 2H). 13C NMR (101 MHz, chloroform-*d*, 300 K) [δ, ppm]: δ 159.06, 139.16, 135.51, 116.63, 110.50, 53.44, 31.30. UV-vis (acetonitrile) [λ_max_/nm, ε/mol^−1^ dm^3^ cm^−1^]: 294, 2.25 × 10^4^; 322, 1.25 × 10^4^; 374, 1.16 × 10^4^; 390, 1.15× 10^4^; 404, 9.92 × 10^3^. IR (powder, cm^−1^): 3082 m br *v*(CH, imine), 2919 m *v*(CH, C*H*_2_-N=CH), 1583s br *v*(C=N). HRMS (*m*/*z*): [M + H]^+^ calcd. for C_13_H_14_N_4_Pd, 333.0333; found, 333.0375.

### 3.4. Spectroscopic pKa Determinations and Solution Species Characterization

The AMT buffer was made up of 100 mM each of Na-acetate, MES, and 200 mM TRIS. The buffer stock solution was prepared and titrated to a pH level of 10 with KOH before being made up to the final volume. The starting titration solution contained 50 μM of ligand, 5% (*v/v*) DMSO, and the buffer was made up to a total volume of 50.0 mL with deionized water. The titration was carried out in a thermostatted titration vessel from Metrohm (37 °C). Specifically, the starting solution was added at a pH level of 10 and a calibrated pH electrode (37 °C) was inserted into the solution. The solution was magnetically stirred and circulated in real time through a 1.0 cm pathlength quartz UV-visible flow cuvette housed within the spectrometer cell holder at 37 °C using a peristaltic pump (Watson-Marlow 120S). The initial spectrum at a pH level of 10 was recorded from 250 nm to 600 nm. Negligible volumes (<0.5 μL) of concentrated HCl (10.2 M) were then carefully diffused via glass capillary into the starting solution to shift the pH level in discrete steps (~0.2 to 0.5 pH units) through the full titration range. After each titrant addition, the pH level was recorded when it had completely stabilized. This was followed by the recording of the UV-visible spectrum of the solution circulating through the flow cuvette.

### 3.5. Fluorescence Spectroscopy

Fluorescence measurements were performed on a JASCO FP-8550 fluorescence spectrophotometer equipped with a Peltier temperature controller. The HSA concentration was kept at 3.0 × 10^−6^ mol dm^−3^ and the excitation and emission slit widths were fixed at 5 and 10 nm, respectively. The concentration of the metal chelates ranged from 0 to 16 × 10^−6^ M. Each complex was made up of DMSO and the final DMSO concentration was under 5% (*v/v*) to prevent the denaturation of HSA. HSA was excited at 295 nm (excitation of Trp-214), and the emission spectra were recorded from 310 to 450 nm. Spectral titrations were carried out at four temperatures (288, 298, and 310 K) in triplicate.

**Correction of fluorescence data.** Inner filter effect (IFE) correction was applied to all fluorescence data using Equation (10) [83],

(10) $$F_{corrected}=F_{observed\;}*10^{Aex*dex+Aem*dem}$$

where *A_ex_* and *A_em_* are the absorbance readings at the excitation and emission wavelengths, while d is the path length of the cuvette.

Further corrections were applied to the concentration of the ligand, i.e., if [Ligand]_added_ was not approximately equal to [Ligand]_free_, the free ligand concentration was calculated using Equation (11) [84]:

(11) $$\;{\left[Ligand\right]}_{free}=\;{\left[Ligand\right]}_{added\;}-\frac{F_{0}}{F_{0}}-\frac{F}{F_{c}}*\;\left[HSA\right]$$

### 3.6. Circular Dichroism (CD) Spectroscopy

Far-UV CD spectra of solutions of HSA (500 × 10^−9^ mol dm^−3^) in the absence and presence of Ni(PrPyrr) and Pd(PrPyrr) (21 µM) were recorded with a JASCO J-1500 CD spectrometer equipped with a Peltier temperature controller (37 °C, 100 mM Na-acetate, 100 mM MES and 200 mM Tris; at pH’s 4, 7 and 9). A scan speed of 100 nm min^−1^ was employed for spectral acquisition with a 0.5 nm data pitch and a response time of 2 s. Each spectrum was the average of three scans. Spectra were recorded over a wavelength range of 200–260 nm (1 cm pathlength quartz cuvette). Due to the presence of a sulfur motif in the MES, we measured the change in HSA’s secondary structure by tracking the change in the molar ellipticity of the protein at 222 nm. [34]

Near UV-vis CD spectra HSA (5.0 × 10^−6^ M; 250–500 nm, 137 °C, 100 mM Na-acetate, 100 mM MES, and 200 mM Tris at pH levels of 4, 7, and 9) were recorded similarly to detect changes in the tertiary structure of HSA. A scan speed of 200 nm min^−1^ was used with a 0.5-nm data pitch (response time, 2 s); each spectrum was the average of three scans from 250 to 310 nm.

UV–CD (ICD) signals of the achiral ligands and metal chelates were induced upon their uptake by HSA. Typically, HSA (15.0 × 10^−6^ M) was incubated with a (15 × 10^−6^ M) of either Ni(PrPyrr) or Pd(PrPyrr) for at least 60 min. A scan speed of 200 nm min^−1^ was used with a 0.5-nm data pitch (response time, 2 s); each spectrum was the average of three scans.

### 3.7. Density Functional Theory

#### 3.7.1. Ligand Optimization

All simulations were performed using Gaussian 16 Rev C.01. [85] Optimization was calculated for H_2_PrPyrr, [H_3_PrPyrr]^+^, [H_4_PrPyrr]^2+^, Pd(PrPyrr), Pd(Cl)(HPrPyrr), [Pd(H_2_O)(HPrPyrr)]^+^, and Pd(OH)(HPrPyrr) at the CAM-B3LYP [86]/DEF2-QZVP [87] level of theory using the GD3BJ [88] empirical dispersion correction. Optimization was performed for Ni(PrPyrr) at the CAM-B3LYP/LANL2DZ [89]/GD3BJ level of theory to ensure square planar geometry for the Ni^II^ metal center. All simulations were carried out in vacuo and GaussView 6.0.16 [90] was used to prepare input files and visualize output data. All jobs finished with normal termination.

#### 3.7.2. Ligand TD-DFT

Time-dependent DFT (TD-DFT) calculations were performed at the CAM-B3LYP/DEF2-QZVP/GD3BJ level of theory to simulate the UV-vis spectra of H_2_PrPyrr, [H_3_PrPyrr]^+^, and [H_4_PrPyrr]^2+^. A water solvent continua (Self-Consistent Reaction Field [91]) was applied and only 20 excited singlet states were computed to give good overlap with experimental spectra.

#### 3.7.3. Macromolecular TD-DFT

Macromolecular TD-DFT ONIOM [80] calculations were performed for ligand-bound HSA to simulate the experimental ICD spectra. The multi-layer method employs a high-level quantum layer computed at the CAM-B3LYP/SDD [92]/GD3BJ level of theory, and a low-level mechanics layer was computed using the UFF [93] force field. The Pd^II^(X) species bound to HSA at all pH levels were calculated to produce 120 excited states (i.e., 60 states per ligand). At pH levels of 4 and 7, only the Pd^II^(X) species were included in the quantum layer. At a pH level of 9, the Pd^II^(X) species as well as Glu-333 were included in the high-level layer. The Ni^II^(X) species bound to HSA were calculated to produce 60 excited states per ligand, except at a pH level of 9, where the macromolecular system HSA•{Ni(PrPyrr)}_2_ was simulated with 160 excited states (i.e., 80 states per ligand). At pH levels of 4 and 7, only the nickel chelates, and organic ligand were included in the quantum layer. At a pH level of 9, Ni(PrPyrr) and residues Lys-195, Trp-214, and Tyr-452 were included in the high-layer while the rest of the protein was treated with the UFF force field. The mechanics layer was given a charge of −13, −6, and +8 at pH levels of 9, 7, and 4, respectively, as calculated after minimization using the OPLS2005 [81] force field.

### 3.8. Molecular Docking & Dynamics

#### 3.8.1. Ligand Preparation

The metal chelates used for docking were first optimized with DFT at the CAM-B3LYP/DEF2-QZVP or the CAM-B3LYP/LANL2DZ level of theory to determine the likely coordination geometry at the metal center and ligand conformation. The DFT-optimized structures were then subjected to optimization using the OPLS2005 force field with standard parameters for LigPrep (LigPrep, Schrödinger, LLC, New York, NY, USA, 2021). This was done to generate files associated with Schrödinger. The force field was not parameterized for the metal chelates under study and it was thus necessary to apply zero-order bonds between the metal ion and the organic framework. The force field then treats the metal-ligand bonds using parameterized electrostatic forces, ignoring the covalent nature of the metal-ligand bond. This is necessary to dock metal complexes using Schrödinger.

#### 3.8.2. Protein Preparation

The X-ray structure of indoxyl sulfate bound to HSA was solved to a final resolution of 2.25 Å and retrieved (PDB: 2BXH [45]) for protein preparation using the Protein Preparation Wizard [94] employed in the Schrödinger Suite 2020-4. The crystal contains two indoxyl sulfate-bound proteins in the unit cell. One protein is selected, and the selection is inverted before deleting all components of the crystal save a single HSA macromolecule. The structure was preprocessed at the relevant pH level (either pH a level of 4, 7, or 9) using Epik [95] to generate heteroatom ionization states. The missing side chains were then added in Maestro. The hydrogen-bond assignment was optimized at the relevant pH level and restrained; minimization was performed using the OPLS2005 force field converging heavy atoms to a root-mean-square deviation (RMSD) of 0.30 Å.

#### 3.8.3. Ligand Docking

The prepared ligands were docked into HSA using Glide [96] to identify potential binding sites for the metal chelates on the transport protein. The receptor grid was centered on Trp-214 with dimensions 40 × 40 × 40 Å^3^ so that most of the protein was sampled for potential binding sites. The portions of the protein not included in the receptor grid are >25 Å away from Trp-214 and are not considered potential binding sites based on the linearity of the Stern–Volmer plots obtained from experimental fluorescent measurements. XP(extra precision) [78] docking was used but standard precision docking may also be employed to identify unknown binding sites because the energy threshold is less strict and may produce poses that do not satisfy the requirements of XP docking. The binding of chelates may be directed to particular subdomains of HSA by imposing positional constraints during grid generation. For example, to direct chelate binding to subdomain IIB, the sulfur atom of Met-329 was selected to define the constraint position and the NOE distance was restricted to between 1.8 and 10 Å. The feature definition for the constraint was applied as the SMARTS pattern for Pd(II), [Pd+2]. These constraints may be applied to any chelate to direct its binding to any subdomain of HSA. Because G-scores produced from docking are poorly correlated to experimental data [79], the G-score is not considered when determining a potential binding site. However, the G-score may be used to discriminate ligand poses within a subdomain (binding site). The docked ligands should corroborate the experimental data, i.e., fluorescence and CD, even if the G-score is low.

#### 3.8.4. Molecular Dynamics

Molecular dynamics (MD) simulations were performed on the best-docked protein-ligand complexes using Desmond [77] and the OPLS2005 force field. The protein–ligand complexes were already pre-processed before the System Builder in Desmond was used to solvate the system with TIP3P [97] water molecules. The biomolecular system was placed in an orthorhombic box with a buffer region of 10 Å between the box boundary and the protein–ligand complex and neutralized with Na^+^ or Cl^−^ ions as necessary. The simulation times were set to 100 ns and the approximate number of frames was kept constant at 1000 so that the recording interval was 100 ps. The model system was relaxed before simulation, and equilibration was performed using the NPT [98] ensemble at 310 K and 1.01 bar. The trajectories were then analyzed in Maestro.

## 4. Conclusions

The interaction of Ni(PrPyrr) and Pd(PrPyrr) with HSA was investigated using complementary spectroscopic techniques at varying pH levels while maintaining a constant ionic strength to understand how pH level influences the uptake of the metal chelates by the protein. Ni(PrPyrr) was prone to demetallation at pH levels of 4 and 7, resulting in [H_3_PrPyrr]^+^ and H_2_PrPyrr being available to bind to HSA along with Ni(PrPyrr), while at a pH level of 9, Ni(PrPyrr) did not undergo hydrolysis. Pd(PrPyrr) had undergone hydrolysis at pH levels of 4, 7, and 9, resulting in Pd(PrPyrr) being in equilibrium with Pd(Cl)(PrPyrr), [Pd(OH_2_)(HPrPyrr)]Cl, or Pd(OH)(HPrPyrr), respectively. Both chelates quenched the intrinsic Trp-214 fluorescence of HSA, via a static quenching mechanism. The Pd^II^(X) chelates had HSA affinity constants (K*_a_* values) that were typical of many small molecule ligands for the protein (10^3^–10^6^ M^−1^) [99,100]. Both Ni^II^ and Pd^II^ chelates bound to HSA with negative ΔH, ΔG, and ΔS values, reflecting a spontaneous enthalpy-driven process governed by London dispersion forces. Glide XP docking simulations in conjunction with fluorescence and induced CD site specificity assays employing probe ligands showed that Ni(PrPyrr) prefers site IB, while the demetallated free ligands H_2_PrPyrr and [H_3_PrPyrr]^+^ prefer Sudlow’s Site I. Pd(PrPyrr) prefers Sudlow’s Site I and its products of hydrolysis prefer subdomain IIB. The far and near UV-CD spectroscopy confirmed that the binding of the ligands minimally perturbs the protein’s secondary structure. Finally, the induced CD spectra recorded for HSA•{Ni^II^(X)} and HSA•{Pd^II^(X)} confirm the uptake of the intact complexes. In summary, we offer a distinctive approach that combines in silico methods with experimental spectroscopic techniques to identify the preferred binding sites of small molecules on HSA.

## Figures and Tables

**Figure 11:**
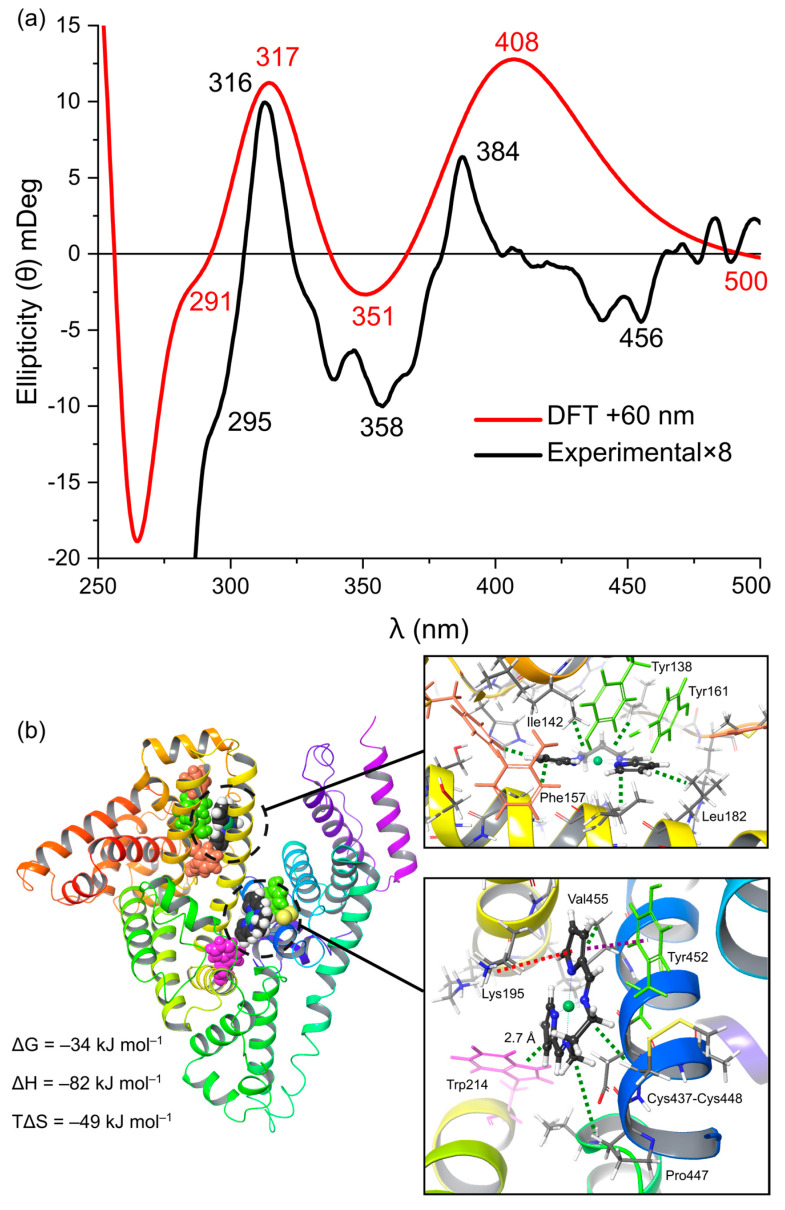
(**a**) Experimental ICD and DFT calculated spectra. **(b)** Ni(PrPyrr) docked into Site 1 and subdomain IB of HSA at a pH level of 9. UV-CD active chromophores Cys–Cys (yellow), Trp (magenta), Tyr (green), and Phe (orange) are shown. The docked protein was simulated with both nickel complexes as well as Lys195, Trp214, and Tyr452 in the quantum layer to produce the calculated spectrum above. The mechanics layer was given a charge of −13 as calculated at a pH level of 9 after minimization using the OPLS [81] force field. The DFT spectrum has been wavelength-corrected and the experimental spectrum has been multiplied by an arbitrary factor to produce the best fit. Ni(PrPyrr) is docked in Site 1 within 2.7 Å of the Trp fluorophore and participates in π- π stacking (purple) with Tyr452, π-cation interactions (red) with Lys195, as well as numerous hydrophobic forces (green). Ni(PrPyrr) docked in subdomain IB is stabilized by hydrophobic forces. These hydrophobic sites within the protein result in ΔS < 0 for the chelate binding event.

**Figure 12:**
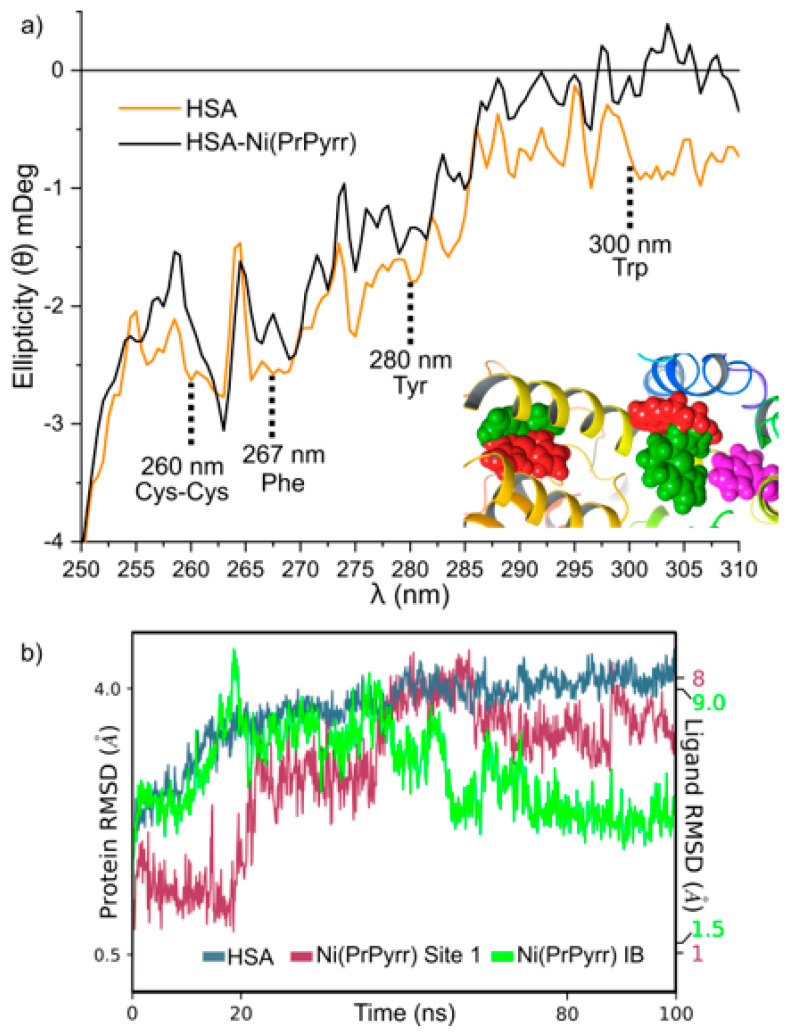
(**a**) Near UV–CD fingerprint region of HSA and HSA•{Ni(PrPyrr)} with 1 equivalent of Ni(PrPyrr) at a pH level of 9. The inset shows the chelates bound to Site 1 and subdomain IB before (red) and after (green) 100 ns molecular dynamics equilibration. Trp-214 is shown in magenta. (**b**) RMSD of HSA and Ni(PrPyrr) docked at Site 1 and subdomain IB over 100 ns. The DFT ICD spectra were calculated before and after MD equilibration with the structure at 0 ns (red) producing the best correlation. The UV–CD shows perturbations at Cys–Cys as well as Trp which are likely due to Ni(PrPyrr) bound at Site 1 within 5 Å of Cys437-Cys448 and Trp-214. Significant perturbations of Phe and Tyr in the presence of Ni(PrPyrr) indicate the chelate is bound at a site rich with these aromatic chromophores. Ni(PrPyrr) docked in subdomain IB is within 5 Å of Phe149, Phe157, Phe165, and Tyr138 and Tyr161.

**Table 1:** Association constants (*K_a_*) for the interaction of Ni^II^(X) and Pd^II^(X) with HSA at different pH levels and temperatures in AMT (100 Na-acetate mM, 100 mM MES, and 200 mM Tris at pH levels 4, 7, and 9).

	T (K)	Ni^(II)^X	Pd^(II)^X
		Log Ka ^[a]^	Log Ka ^[a]^
pH 4	288	4.62 (±0.01)	5.15 (±0.02)
	298	3.65 (±0.06)	4.62 (±0.01)
	310	2.22 (±0.06)	3.71 (±0.04)
	288	4.43 (±0.03)	5.55 (±0.04)
pH 7	298	4.18 (±0.04)	5.13 (±0.12)
	310	3.29 (±0.02)	4.93 (±0.12)
	288	6.42 (±0.04)	5.74 (±0.12)
pH 9	298	5.81 (±0.01)	5.31 (±0.10)
	310	5.12 (±0.04)	4.97 (±0.06)

^[a]^ The estimated standard deviations of the least significant digits are given in parentheses.

**Table 2:** Thermodynamic parameters for the binding of Ni^II^(X) and Pd^II^(X) by HSA at different pH levels in AMT buffer (100 Na-acetate mM, 100 mM MES, and 200 mM Tris at pH levels of 4, 7, and 9).

			Ni^II^(X) ^[a]^			Pd^II^(X) ^[b]^	
pH	T (K)	*∆G* ^[c]^ kJ mol^−1^	*∆H* ^[c]^ kJ mol^−1^	*T∆S* ^[c]^ kJ mol^−1^	*∆G* ^[c]^ kJ mol^−1^	*∆H* ^[c]^ kJ mol^−1^	*T∆S* ^[c]^ kJ mol^−1^
	288.00	−25.26 (±0.9)			−31.31 (±0.1)		
4.00	298.00	−20.79 (±0.9)	−154 (±3.6)	−133 (±4.5)	−28.30 (±0.1)	−118 (±27)	−89.70 (±11)
	310.00	−15.43 (±0.9)			−24.69 (±0.1)		
	288.00	−24.44 (±0.7)			−30.43 (±0.1)		
7.00	298.00	−23.91 (±0.7)	−39.70 (±0.3)	−15.79 (±3)	−29.74 (±0.1)	−50.30 (±1)	−20.56 (±0.1)
	310.00	−23.27 (±0.7)			−28.91 (±0.1)		
	288.00	−35.46 (±0.1)			−31.75 (±0.4)		
9.00	298.00	−33.83 (±0.1)	−82.40 (±12)	−48.57 (±12)	−30.46 (±0.4)	−68.90 (±10)	−38.44 (±10.4)
	310.00	−31.87 (±0.1)			−28.91 (±0.4)		

^[a]^ Ni^II^(X) = Ni(PrPyrr) + X. ^[b]^ Pd^II^(X) = Pd(Cl)(PrPyrr), [Pd(OH_2_)(HPrPyrr)]Cl, or Pd(OH)(HPrPyrr). ^[c]^ The estimated standard deviations of the least significant digits are given in parentheses.

## Data Availability

Available upon request.

## Supplementary Materials

The following supporting information can be downloaded at: https://www.mdpi.com/article/10.3390/molecules28227466/s1, Figure S1: Trp–214 fluorescence emission maximum dependence on pH.; Figure S2: Normalized fluorescence emission maximum of native HSA and (a) HSA•{Ni^II^(X)} and HSA•{Pd^II^(X)} at pH’s 4, 7 and 9. Upon binding of Ni^II^(X) to HSA. Figure S3: Plots of the far-UV CD spectra of native HSA and the protein incubated with saturating doses of (a) Ni^II^(X) and (b) Pd^II^(X). Figures S4 and S5 are the TD-DFT UV-vis simulations of the free and protonated ligands. Figures S6–S15 represent the calculated ICD, TD-DFT and near UV-CD fingerprint regions of Ni^II^(X) and Pd^II^(X) binding to and interacting with HSA. Figure S16: HSA docked with Pd^II^(X) at pH 4, 7 and 9. Table S1: Stern-Volmer quenching constants ($K_{SV}$), bimolecular quenching rate constants ($K_{q}$), for the interaction of Ni^II^(X) and Pd^II^(X) with HSA at different temperatures in AMT buffer at pH’s 4, 7 and 9.
