# Supplementary material for: Spectroscopic and Computational pH Study of NiII and PdII Pyrrole-Imine Chelates with Human Serum Albumin
Source: Molecules. 2023 Nov 7;28(22):7466. doi: 10.3390/molecules28227466 (PMC10673405; doi:10.3390/molecules28227466)
Supplement: Supplementary file 1 [file molecules-28-07466-s001.zip › molecules-2674043-supplementary.pdf]

Supporting Information:

Spectroscopic and computational pH study of Ni^{II} and Pd^{II} Pyrrole-imine chelates with human serum albumin

Sheldon Sookai[1]*, Matthew Bracken[1] and Monika Nowakowska[1]

[1] Molecular Sciences Institute, School of Chemistry, University of the Witwatersrand, PO WITS 2050, Johannesburg, South Africa

Email address: [1]Sheldon.sookai@wits.ac.za

[1]matthewbracken125@gmail.com

[1]monika.nowakowska@wits.ac.za

This supporting information contains 16 Figures and 1 table.

1. Figures

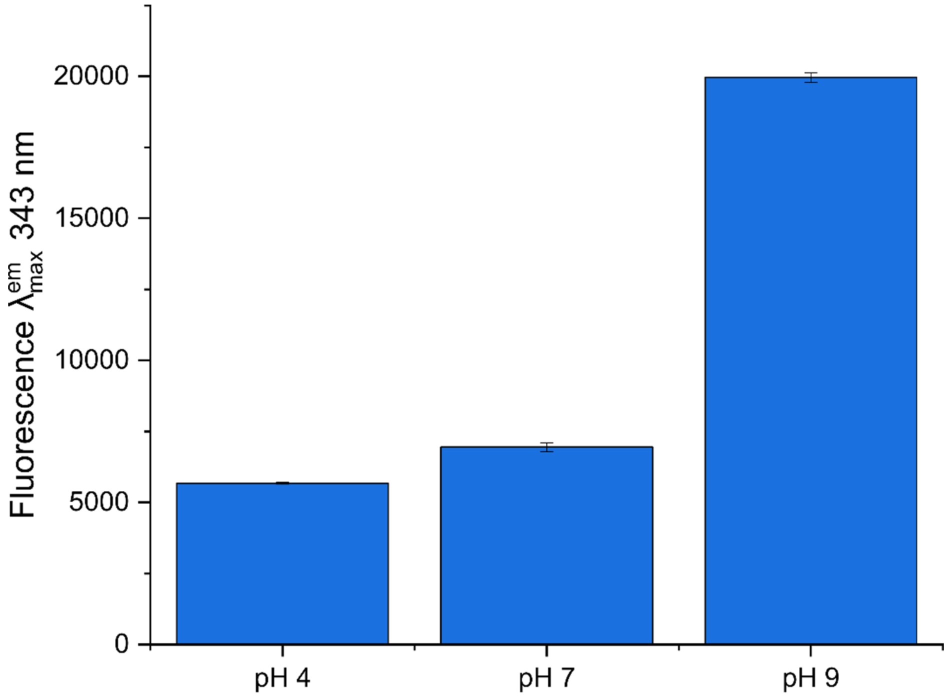

Fig. S1 Trp–214 fluorescence emission maximum dependence on pH. Error bars indicate the standard deviation of three independent titrations.

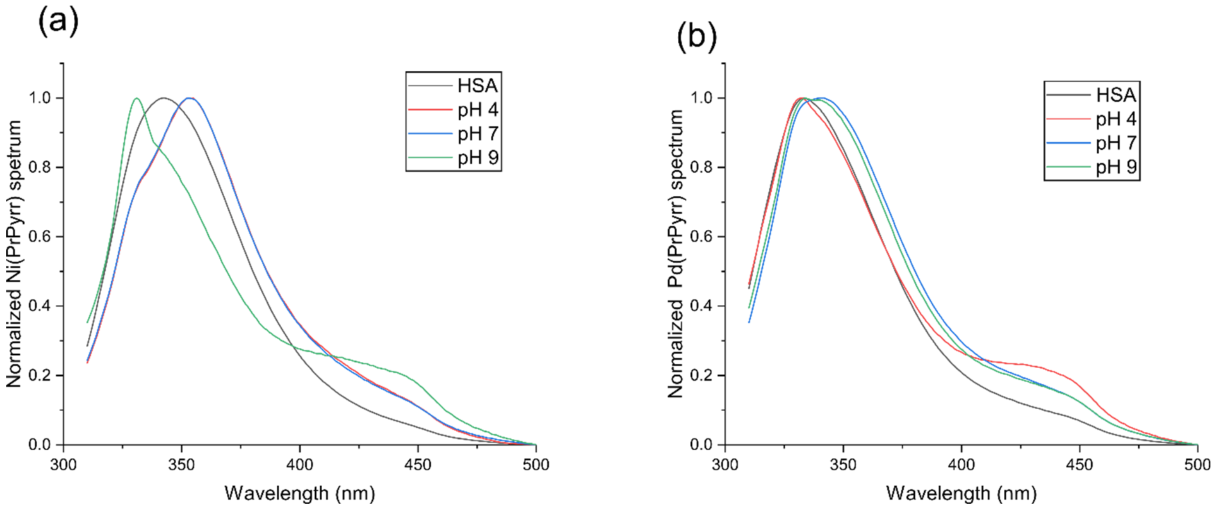

Fig. S2 Normalized fluorescence emission maximum of native HSA and (a) HSA•{Ni^{II}(X)} and HSA•{Pd^{II}(X)} at pH's 4, 7 and 9. Upon binding of Ni^{II}(X) to HSA. The complex induced red shifts at pH 4 and 7 and a blue shift at pH 9, while Pd^{II}(X) binding to HSA induced no emission maximum shift at pH 4 but red shifts at pH 7 and 9.

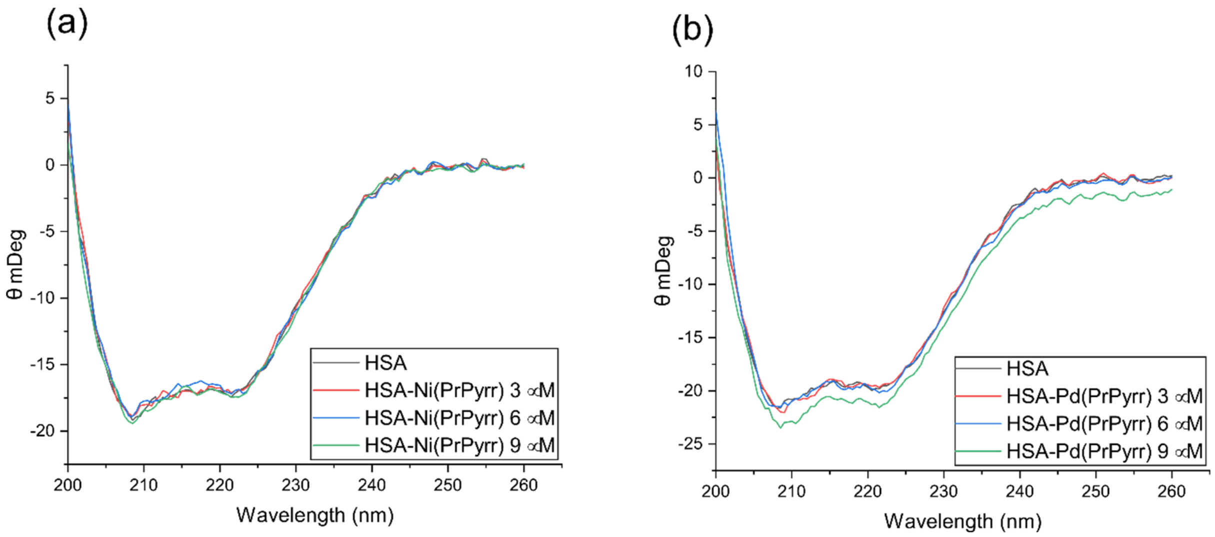

Fig. S3 Plots of the far-UV CD spectra of native HSA and the protein incubated with saturating doses of (a) Ni^{II}(X) and (b) Pd^{II}(X) recorded at 298 K in 50 mM KH_2PO_4 buffer at pH 7.50. (In each case the X represents the ligand combinations of the equilibrium species.) The ligand dose ranged from 3 to 9 μM.

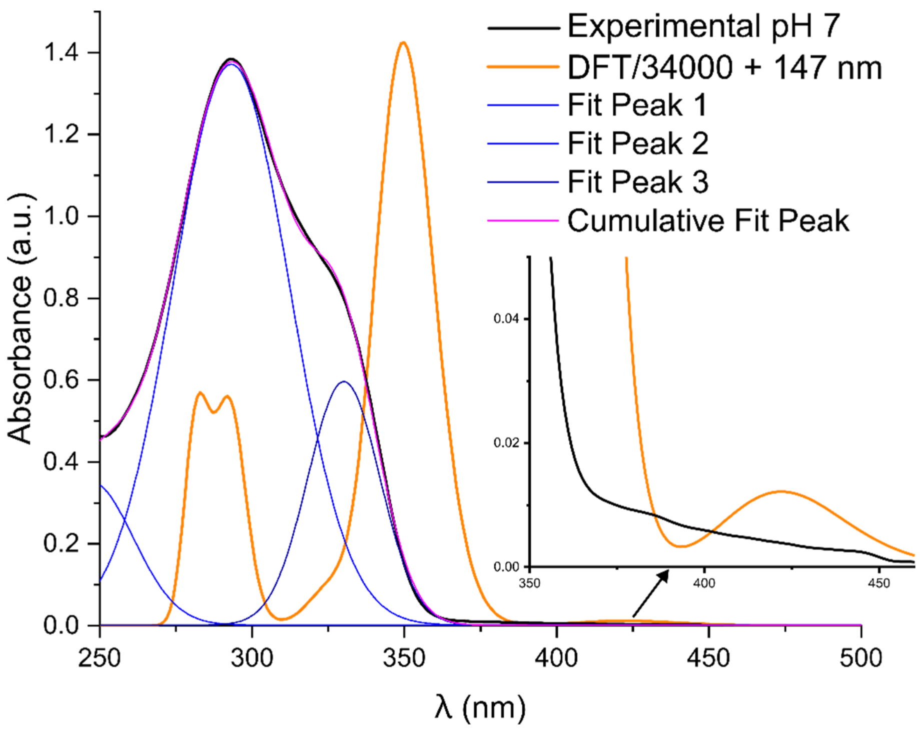

Fig. S4 UV-vis absorbance spectrum of H_2PrPyrr in AMT buffer at pH 7 and 25 °C. TD-DFT calculations were used to predict the protonation state of H_2PrPyrr at pH 7 in aqueous media. H_2PrPyrr was simulated in its neutral state using a water solvent model and the calculated spectrum shows good agreement with experimental data. Peak deconvolution was performed for the experimental data over the wavelength range 250-350 nm using a Gauss model in Origin Pro 2022. Three peaks were fitted with maxima at 250, 296, and 332 nm. These correspond to DFT calculated peaks at 284, 294, and 351 nm. The inset shows a shoulder in the experimental spectrum circa 400 nm, which has been calculated at 423 nm. These data, along with the experimental *pKa,* strongly suggest that H_2PrPyrr is the dominant species at pH 7.

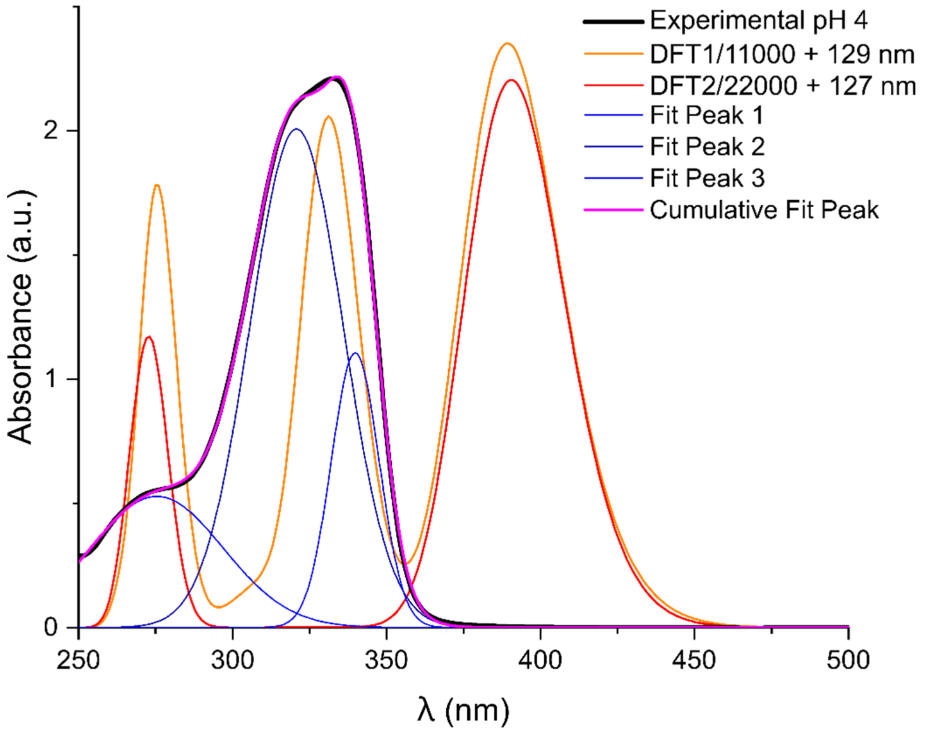

Fig. S5 UV-vis absorbance spectrum of $[H_3PrPyrr]^+$ in AMT buffer at pH 4 and 25 °C. TD-DFT calculations were used to predict the protonation state of $H_2PrPyrr$ at pH 4 in aqueous media. $[H_3PrPyrr]^+$ and $[H_4PrPyrr]^{2+}$ were simulated and have been labelled DFT1 (red) and DFT2 (orange) respectively. Peak deconvolution was performed for the experimental data using a Gauss model in Origin Pro 2022. Three peaks were fitted to the experimental data with maxima at 275, 322, and 337 nm. These correspond to DFT calculated peaks for $[H_3PrPyrr]^+$ at 275, 334, and 393 nm. The DFT calculation for $[H_4PrPyrr]^{2+}$ only shows two peaks at 270nm and 394 nm. These data, along with the experimental *pKa*, strongly suggest that $[H_3PrPyrr]^+$ is the dominant species at pH 4.

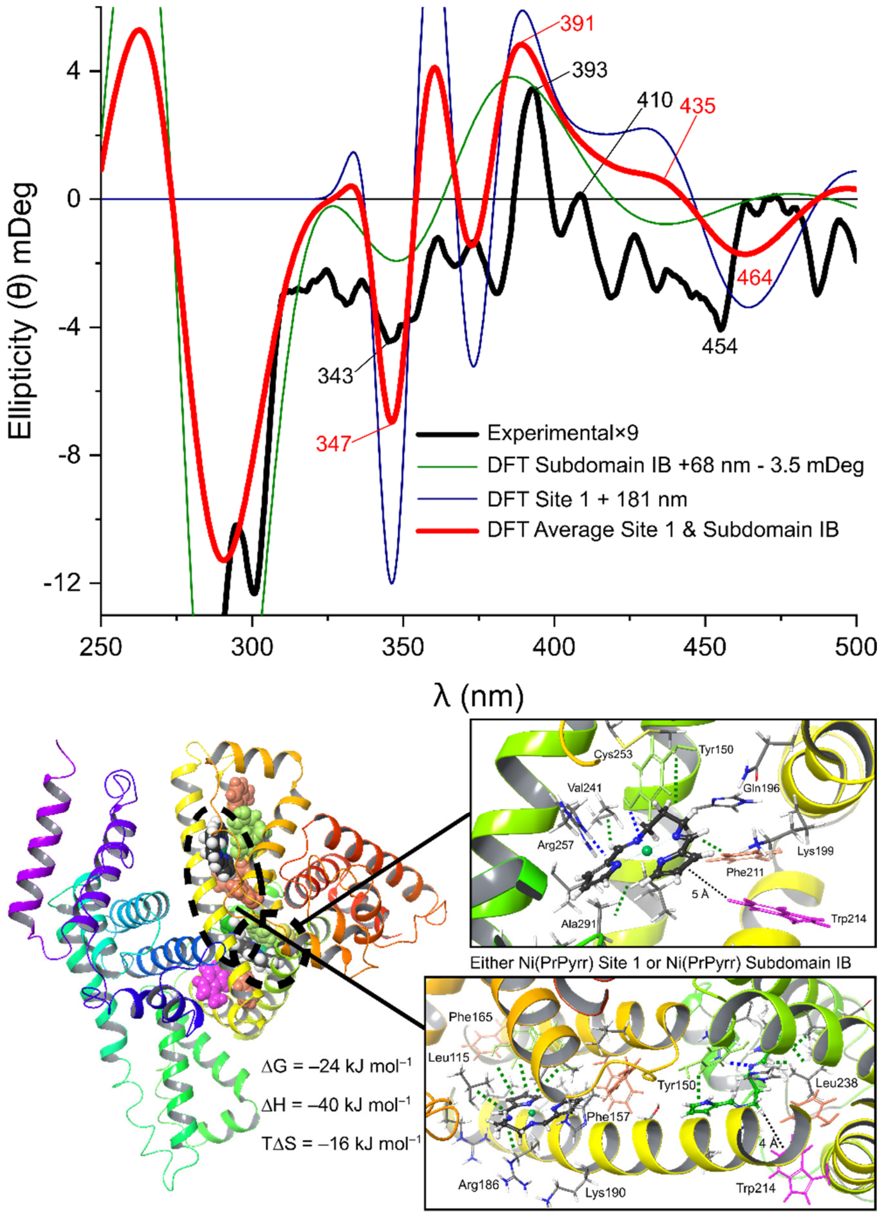

Fig. S6 Top: Experimental ICD and DFT calculated spectra. Bottom: Ni(PrPyrr) either docked into Site 1 or subdomain IB of HSA at pH 7. UV-CD active chromophores Cys-Cys (yellow), Trp (magenta), Tyr (green), and Phe (orange) are shown. Due to significant demetallation of Ni(PrPyrr) in the AMT buffer at pH 7, the free ligand H_2PrPyrr is present at high concentrations and available to bind Site 1 of HSA. When the free base is bound, it leaves Site 1 unavailable for chelate binding and Ni(PrPyrr) is directed to subdomain IB. Hence, a species distribution exists where HSA•{Ni(PrPyrr)} and HSA•{H_2PrPyrr}•{Ni(PrPyrr)} are both present in solution. As a result, the DFT calculated spectra average for each species produces the best correlation with the experimental spectrum. When Ni(PrPyrr) is docked at Site 1, it is within 5 Å of Trp214 and stabilized by hydrophobic (green) and polar (blue) forces. When H_2PrPyrr is docked at Site 1, it is within 4 Å of Trp214 and stabilized by hydrophobic and polar forces while Ni(PrPyrr) bound to subdomain IB is stabilized by numerous hydrophobic interactions to residues including Leu155 and Phe165. The docked protein was simulated with only the chelates in quantum layer and the mechanics layer was given a charge of –6 after optimization at pH 7.

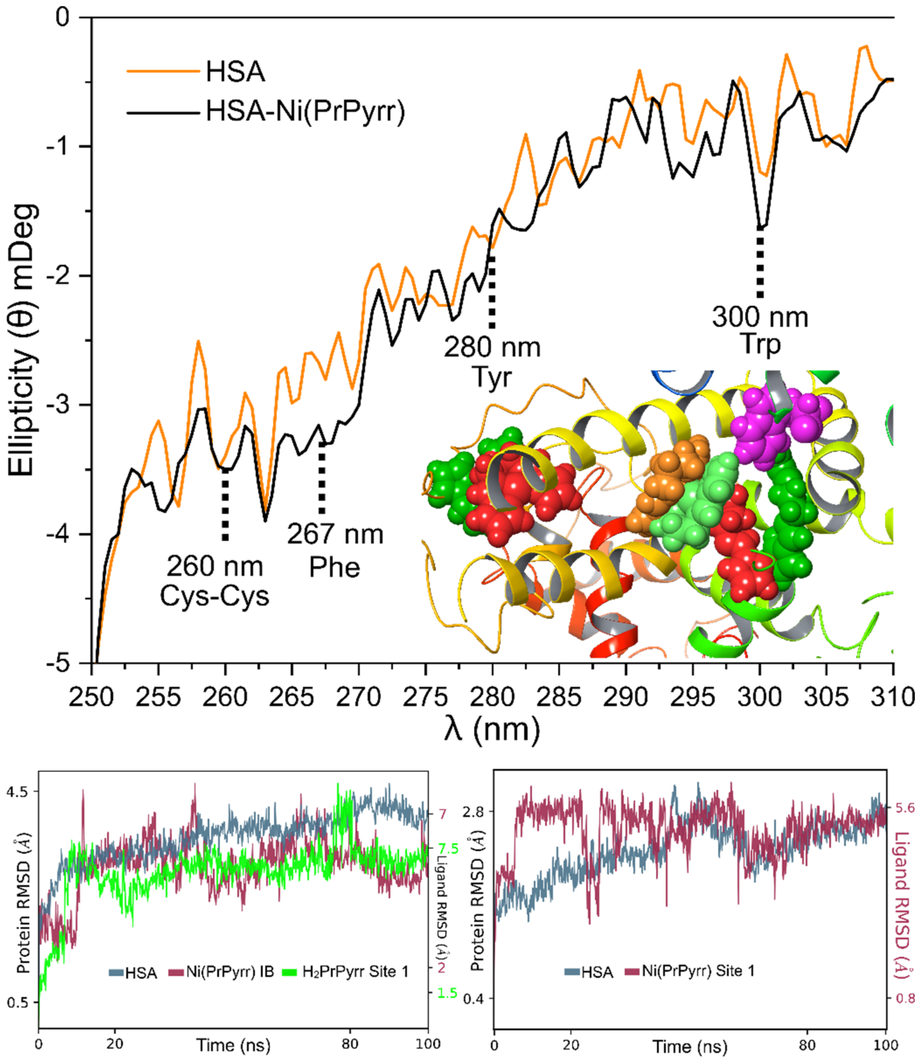

Fig. S7 Top: Near UV-CD fingerprint region of HSA and HSA•{Ni(PrPyrr)} with 1 equivalent of Ni(PrPyrr) at pH 7. The inset shows the free ligand bound to Site 1 and Ni(PrPyrr) bound to subdomain IB before (red) and after (green) 100 ns molecular dynamics equilibration. The second species present is Ni(PrPyrr) bound at Site 1 before (orange) and after (light-green) 100 ns MD equilibration. Trp214 is shown in magenta. Bottom: RMSD of HSA and Ni(PrPyrr) docked at Site 1 and subdomain IB, as well as the free ligand H_2PrPyrr over 100 ns. The DFT ICD spectra were calculated before and after MD equilibration with the structure at 0 ns (red/orange) producing the best correlation. The UV-CD shows small perturbations at Cys-Cys likely due to Ni(PrPyrr) bound in Site 1 within 5 Å of Cys245-Cys253. When the free base H_2PrPyrr is bound in Site 1 and the nickel chelate is bound at subdomain IB, these ligands are > 5 Å away from any disulfide moiety resulting in minimal perturbation of this chromophore. Hence, due to small changes at 260 nm, it seems like that HSA•{H_2PrPyrr }•{Ni(PrPyrr)} is the dominant species in solution. The remaining aromatic perturbations of Trp, Tyr, and Phe are due to either the free ligand or chelate bound in Site 1 as well as Ni(PrPyrr) bound in Phe and Tyr rich subdomain IB.

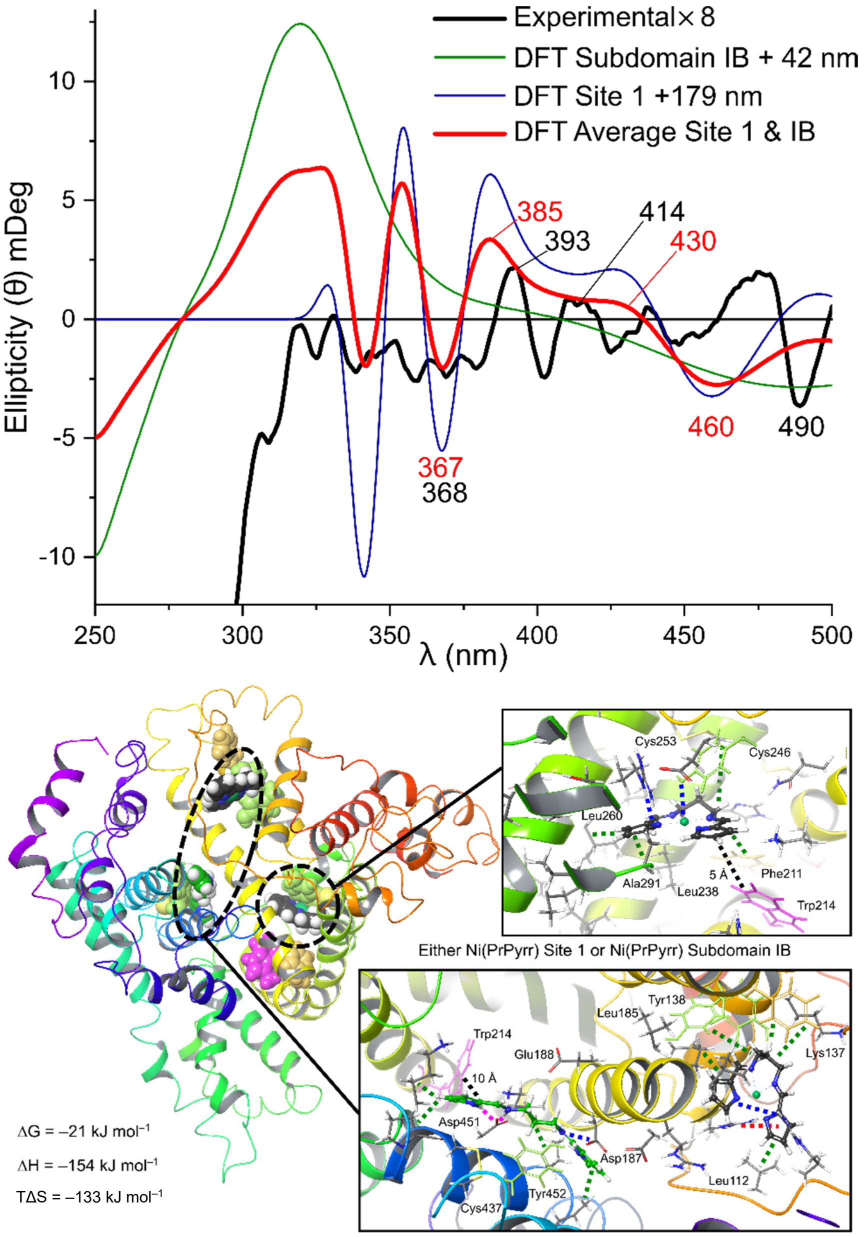

Fig. S8 Top: Experimental ICD and DFT calculated spectra. Bottom: Ni(PrPyrr) either docked into Site 1 or subdomain IB of HSA at pH 4. UV-CD active chromophores Cys-Cys (yellow), Trp (magenta), Tyr (green), and Phe (orange) are shown. Due to significant demetallation of Ni(PrPyrr) in the AMT buffer at pH 4, the free ligand $[H_3PrPyrr]^+$ is present at high concentrations and available to bind Site 1 of HSA. When the free base is bound, it leaves Site 1 unavailable for chelate binding and Ni(PrPyrr) is directed to subdomain IB. Hence, a species distribution exists where HSA•{Ni(PrPyrr)} and HSA•{$[H_3PrPyrr]^+$}•{Ni(PrPyrr)} are both present in solution. As a result, the DFT calculated spectra average for each species produces the best correlation with the experimental spectrum. When Ni(PrPyrr) is docked at Site 1, it is within 5 Å of Trp214 and stabilized by hydrophobic (green) and polar (blue) forces. When $[H_3PrPyrr]^+$ is docked at Site 1, it is within 10 Å of Trp214 and stabilized by hydrophobic and polar forces as well as hydrogen-bonding (pink) between the protonated imine and Asp451. Ni(PrPyrr) bound to subdomain IB is stabilized by numerous hydrophobic interactions to residues including Leu185 and Tyr138 as well as π-cation interactions (red). The docked protein was simulated with only the chelates and $[H_3PrPyrr]^+$ in the quantum layer and the mechanics layer was given a charge of +8 after optimization at pH 4.

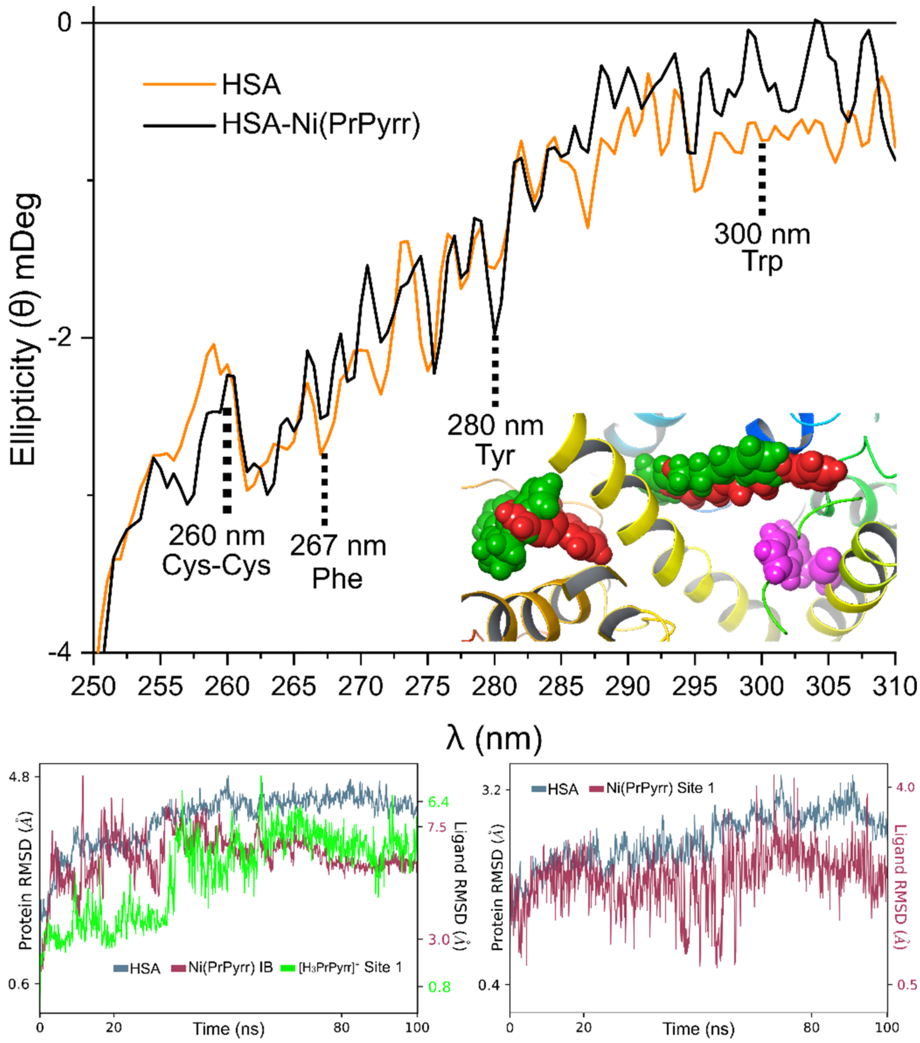

Fig. S9 Top: Near UV-CD fingerprint region of HSA and HSA•{Ni(PrPyrr)} with 1 equivalent of Ni(PrPyrr) at pH 4. The inset shows the free ligand bound to Site 1 and Ni(PrPyrr) bound to subdomain IB before (red) and after (green) 100 ns molecular dynamics equilibration. Trp214 is shown in magenta. Ni(PrPyrr) bound to subdomain IB not shown in inset. Bottom: RMSD of HSA and Ni(PrPyrr) docked at Site 1 and subdomain IB, as well as the free ligand $[H_3PrPyrr]^+$ over 100 ns. The DFT ICD spectra were calculated before and after MD equilibration with the structure at 100 ns (green) producing the best correlation. The UV-CD shows perturbations at Cys-Cys likely due to $[H_3PrPyrr]^+$ bound in Site 1 within 5 Å of Cys437-Cys448. Furthermore, Ni(PrPyrr) docked in Site 1 is within 5 Å of Cys245-Cys253 as well as Cys200-Cys246. Hence, either species HSA•{Ni(PrPyrr)} and HSA•{$[H_3PrPyrr]^+$}•{Ni(PrPyrr)} will result in perturbations at 260 nm. The remaining aromatic perturbations of Trp, Tyr, and Phe are due to either the free ligand or chelate bound in Site 1 within 10 Å of Trp as well as Ni(PrPyrr) bound in Phe and Tyr rich subdomain IB.

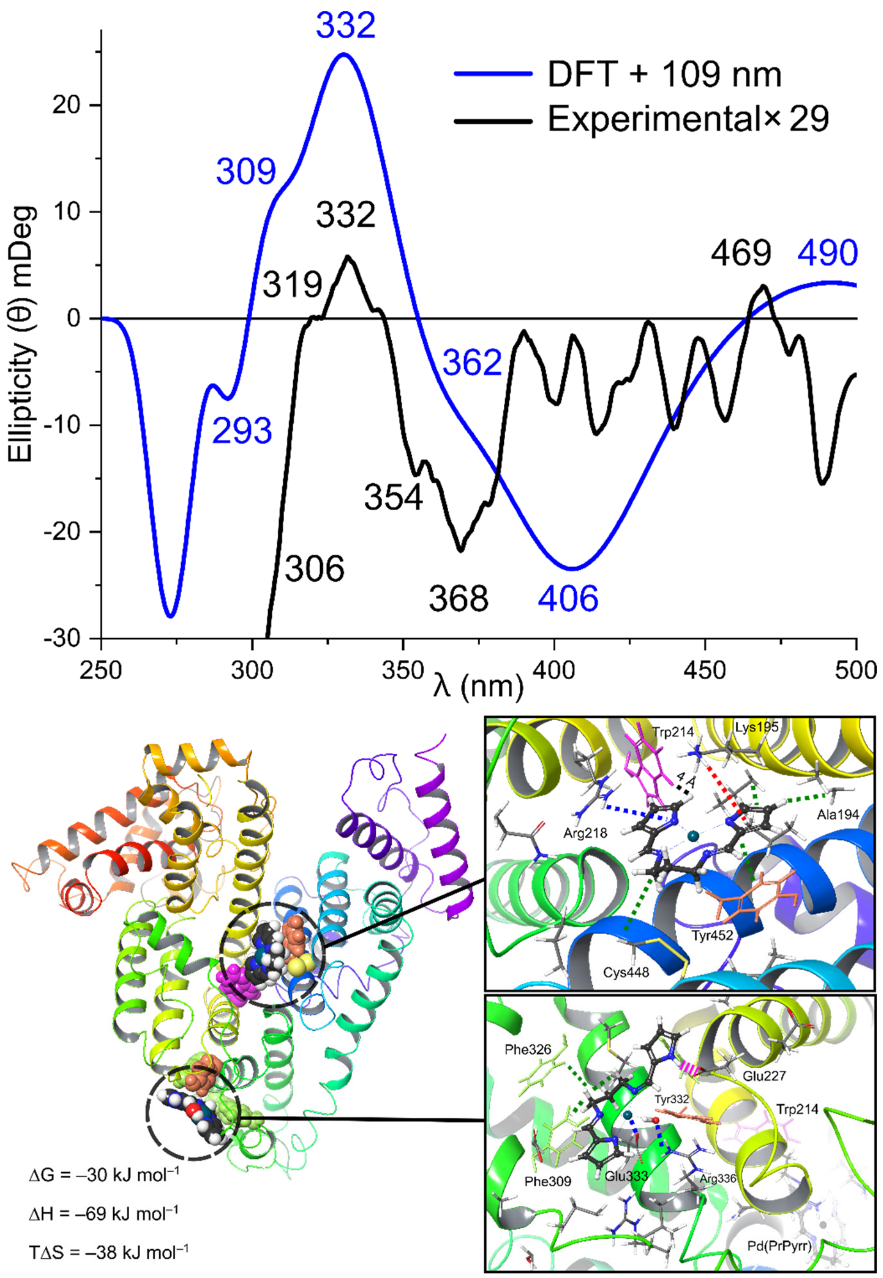

Fig. S10 Top: Experimental ICD and DFT calculated spectra. Bottom: Pd(PrPyrr) docked into Site 1 and Pd(OH)(HPrPyrr) subdomain IIB of HSA at pH 9. UV-CD active chromophores Cys-Cys (yellow), Trp (magenta), Tyr (green), and Phe (orange) are shown. The docked protein was simulated with both palladium complex species as well as Glu333 in the quantum layer to produce the calculated spectrum above. The mechanics layer was given a charge of –13 as calculated at pH 9 after minimization using the OPLS force field. The DFT spectrum has been wavelength-corrected and the experimental spectrum multiplied by an arbitrary factor to produce the best fit. Pd(PrPyrr) is docked in Site 1 within 4 Å of the Trp fluorophore and participates in π-cation interactions (red) with Lys195, as well as numerous hydrophobic forces (green) and polar interactions (blue) with Arg218. Pd(OH)(HPrPyrr) docked in subdomain IIB is stabilized by hydrophobic interactions between Phe326 and Phe309, as well as hydrogen-bonding (pink) between the pyrrole and Glu227. The polar forces (blue) between Glu333 and the Pd^{2+} ion stabilize at a distance of 2.2 Å. These hydrophobic sites within the protein result in $\Delta S < 0$ for the chelate binding event while London dispersion forces, hydrogen-bonding, and π-cation interactions result in a favorable ΔH.

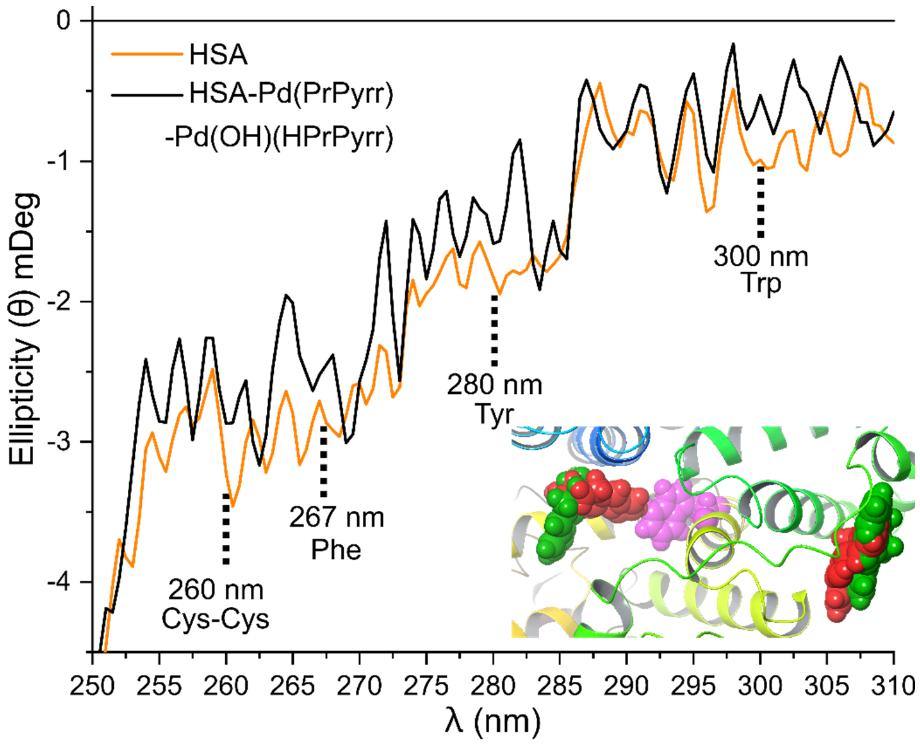

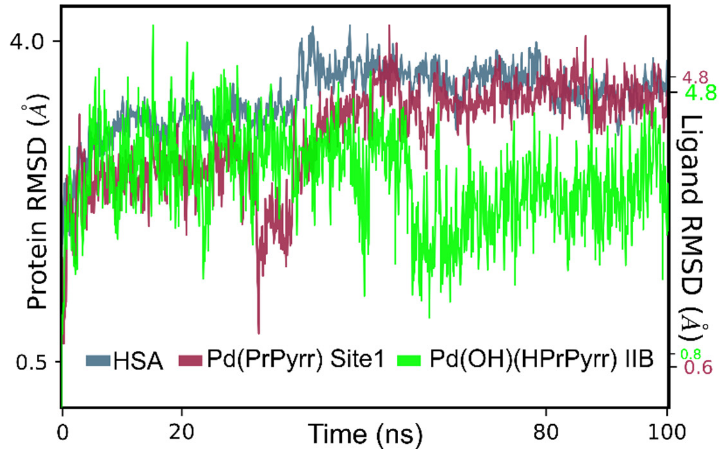

Fig. S11 Top Near UV-CD fingerprint region of HSA and HSA•{Pd(PrPyrr)}•{ Pd(OH)(HPrPyrr)} with 1 equivalent of Pd^{II}(X) at pH 9. The inset shows Pd(PrPyrr) bound to Site 1 and Pd(OH)(HPrPyrr) bound at subdomain IIB before (red) and after (green) 100 ns molecular dynamics equilibration. Trp214 is shown in magenta. Bottom: RMSD of HSA and Pd(PrPyrr) docked at Site 1 and Pd(OH)(HPrPyrr) docked at subdomain IIB over 100 ns. The DFT ICD spectra were calculated before and after MD equilibration with the structure at 0 ns (red) producing the best correlation. The UV-CD shows significant perturbations at Cys-Cys as well as Trp which are likely due to Pd(PrPyrr) bound at Site 1 within 5 Å of Cys437-Cys448 and Trp214. Significant perturbations of Phe and Tyr in the presence of Pd^{II}(X) indicate the chelate is bound at a site rich with these aromatic chromophores. Pd(OH)(HPrPyrr) docked in subdomain IIB is within 5 Å of Phe228, Phe309, Phe326 and Tyr332.

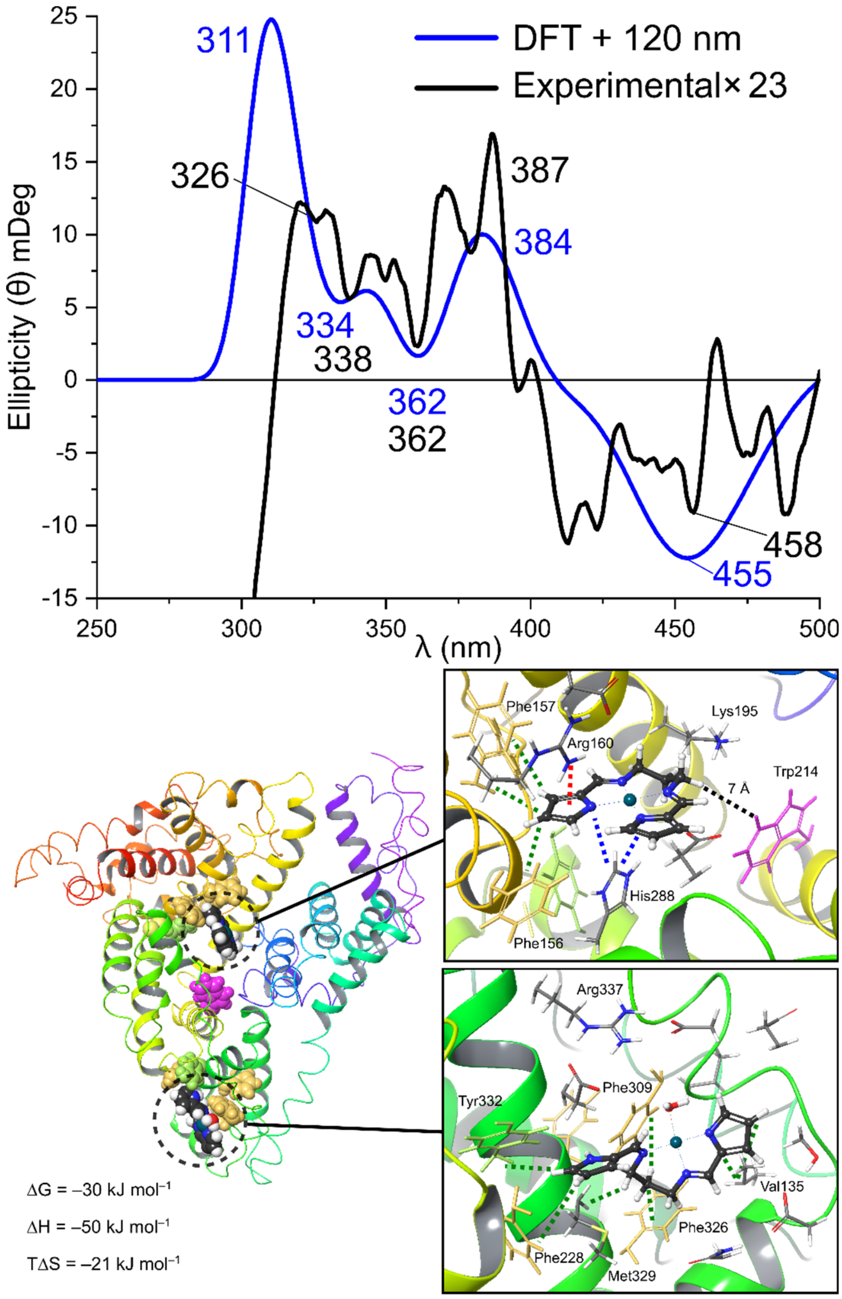

Fig. S12 Top: Experimental ICD and DFT calculated spectra. Bottom: Pd(PrPyrr) docked into Site 1 and $[Pd(OH_2)(HPrPyrr)]^+$ docked at subdomain IIB of HSA at pH 7. UV-CD active chromophores Cys-Cys (yellow), Trp (magenta), Tyr (green), and Phe (orange) are shown. The docked protein was simulated with both palladium complex species in the quantum layer while the mechanics layer was given a charge of –6 to produce the calculated spectrum above. The DFT spectrum has been wavelength-corrected and the experimental spectrum multiplied by an arbitrary factor to produce the best fit. Pd(PrPyrr) is docked in Site 1 within 7 Å of the Trp fluorophore and participates in π-cation interactions (red) with Arg160, as well as numerous hydrophobic interactions (green) with Phe156 and Phe157. Polar forces (blue) between His288 and the ligand are shown. $[Pd(OH_2)(HPrPyrr)]^+$ docked in subdomain IIB is stabilized by hydrophobic interactions between Val135 and Met329 as well as various phenylalanine residues. These hydrophobic sites within the protein result in ΔS < 0 for the chelate binding event while predominately London dispersion forces result in a favorable ΔH.

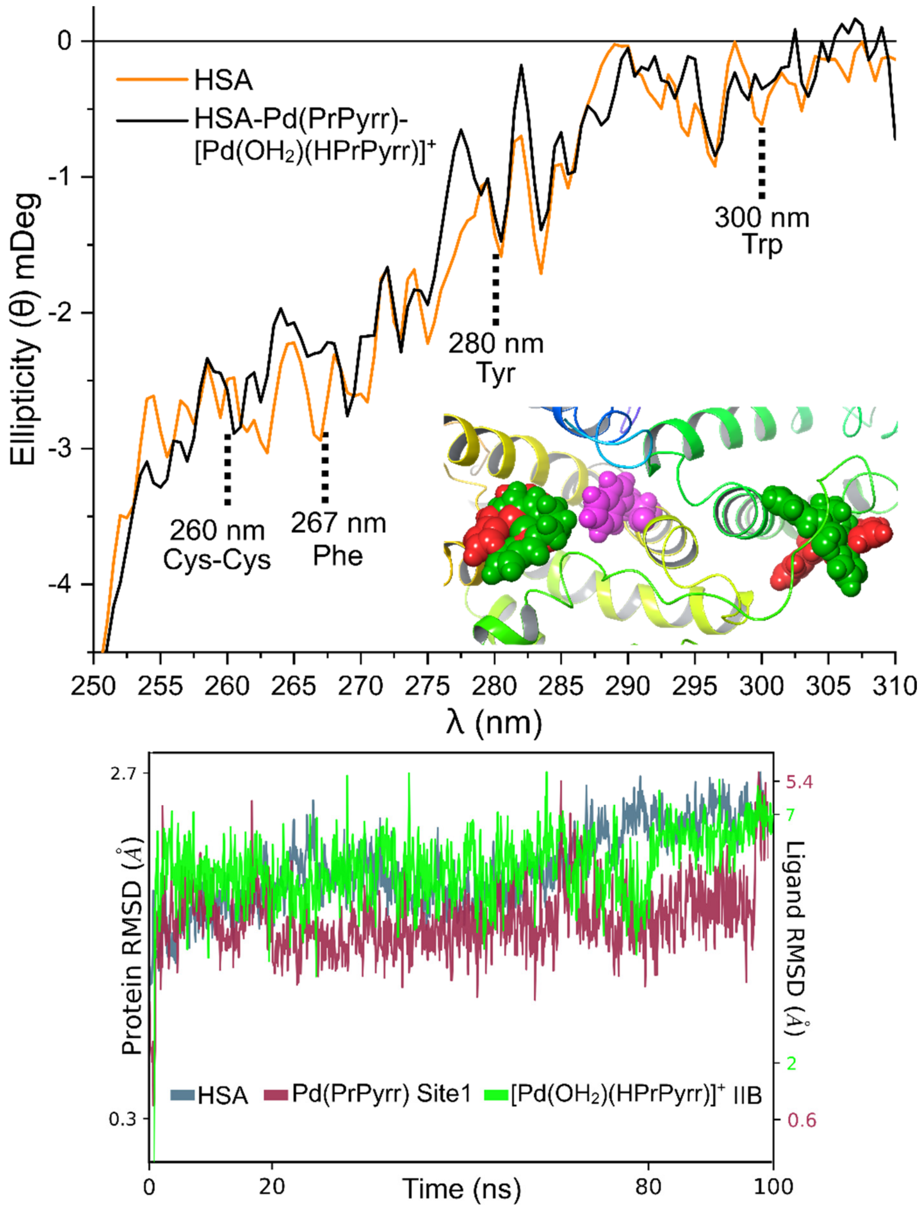

Fig. S13 Top: Near UV-CD fingerprint region of HSA and HSA•{Pd(PrPyrr)}•{[Pd(OH$_2$)(HPrPyrr)]$^+$} with 1 equivalent of Pd^{II}(X) at pH 7. The inset shows Pd(PrPyrr) bound to Site 1 and [Pd(OH$_2$)(HPrPyrr)]$^+$ bound at subdomain IIB before (red) and after (green) 100 ns molecular dynamics equilibration. Trp214 is shown in magenta. Bottom: RMSD of HSA and Pd(PrPyrr) docked at Site 1 and [Pd(OH$_2$)(HPrPyrr)]$^+$ docked at subdomain IIB over 100 ns. The DFT ICD spectra were calculated before and after MD equilibration with the structure at 0 ns (red) producing the best correlation. The UV-CD shows perturbations at Cys-Cys which are likely due to Pd(PrPyrr) bound at Site 1 within 10 Å of Cys278-Cys289 and Cys437-Cys448. This is in contrast to the significant perturbations of Cys-Cys in the presence of Pd^{II}(X) at pH 9 where Pd(PrPyrr) is within 5 Å of Cys437-Cys448. Additionally, while Trp214 is perturbed by Pd(PrPyrr) at pH 7, the changes at pH 9 are greater and reflect the distance between Pd(PrPyrr) and Trp214 (7 Å at pH 7 and 4 Å at pH 9). Phe and Tyr are also significantly disturbed between 255 and 285 nm when Pd^{II}(X) is present, indicating the binding site is rich with these aromatic chromophores. [Pd(OH$_2$)(HPrPyrr)]$^+$ docked in subdomain IIB is within 5 Å of Phe228, Phe309, Phe326 and Tyr332 while Pd(PrPyrr) docked at Site 1 is stabilized by hydrophobic interactions to Phe149, Phe156, and Phe157.

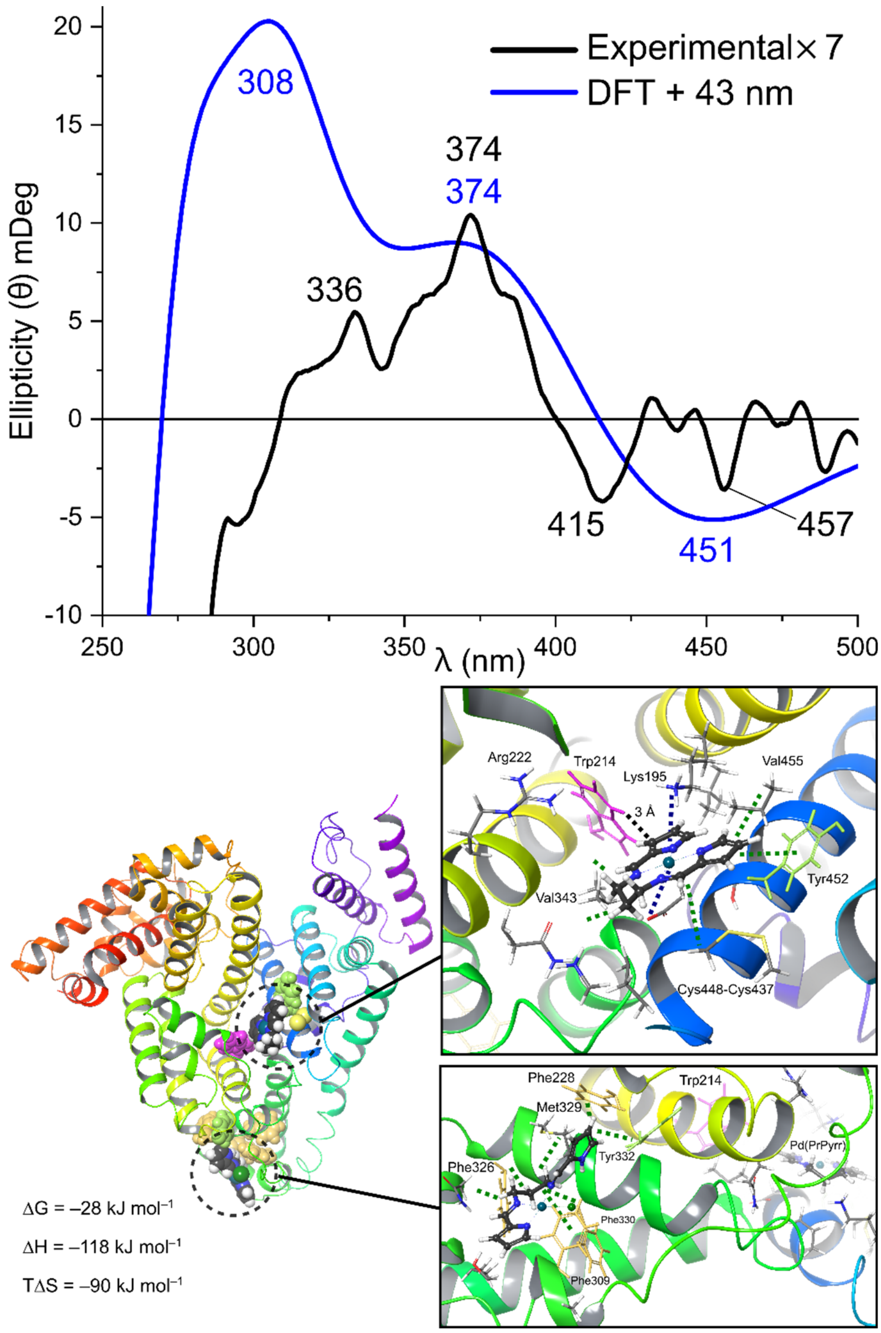

Fig. S14 Top: Experimental ICD and DFT calculated spectra. Bottom: Pd(PrPyrr) docked into Site 1 and Pd(Cl)(HPrPyrr) docked at subdomain IIB of HSA at pH 4. UV-CD active chromophores Cys-Cys (yellow), Trp (magenta), Tyr (green), and Phe (orange) are shown. The docked protein was simulated with both palladium complex species in the quantum layer while the mechanics layer was given a charge of +12 to produce the calculated spectrum above. The DFT spectrum has been wavelength-corrected and the experimental spectrum multiplied by an arbitrary factor to produce the best fit. Pd(PrPyrr) is docked in Site 1 within 3 Å of the Trp fluorophore and participates in numerous hydrophobic interactions (green) with valine, tyrosine, and cysteine which make up the hydrophobic binding site. Polar forces (blue) between the ligand and Lys195 are shown as well as between Pd^{2+} and Asp451 with a metal-to-residue distance of 5 Å. Pd(Cl)(HPrPyrr) docked in subdomain IIB is stabilized by hydrophobic interactions between Met329 and Tyr332 as well as various phenylalanine residues. These hydrophobic sites within the protein result in ΔS < 0 for the chelate binding event while predominately London dispersion forces result in a favourable ΔH.

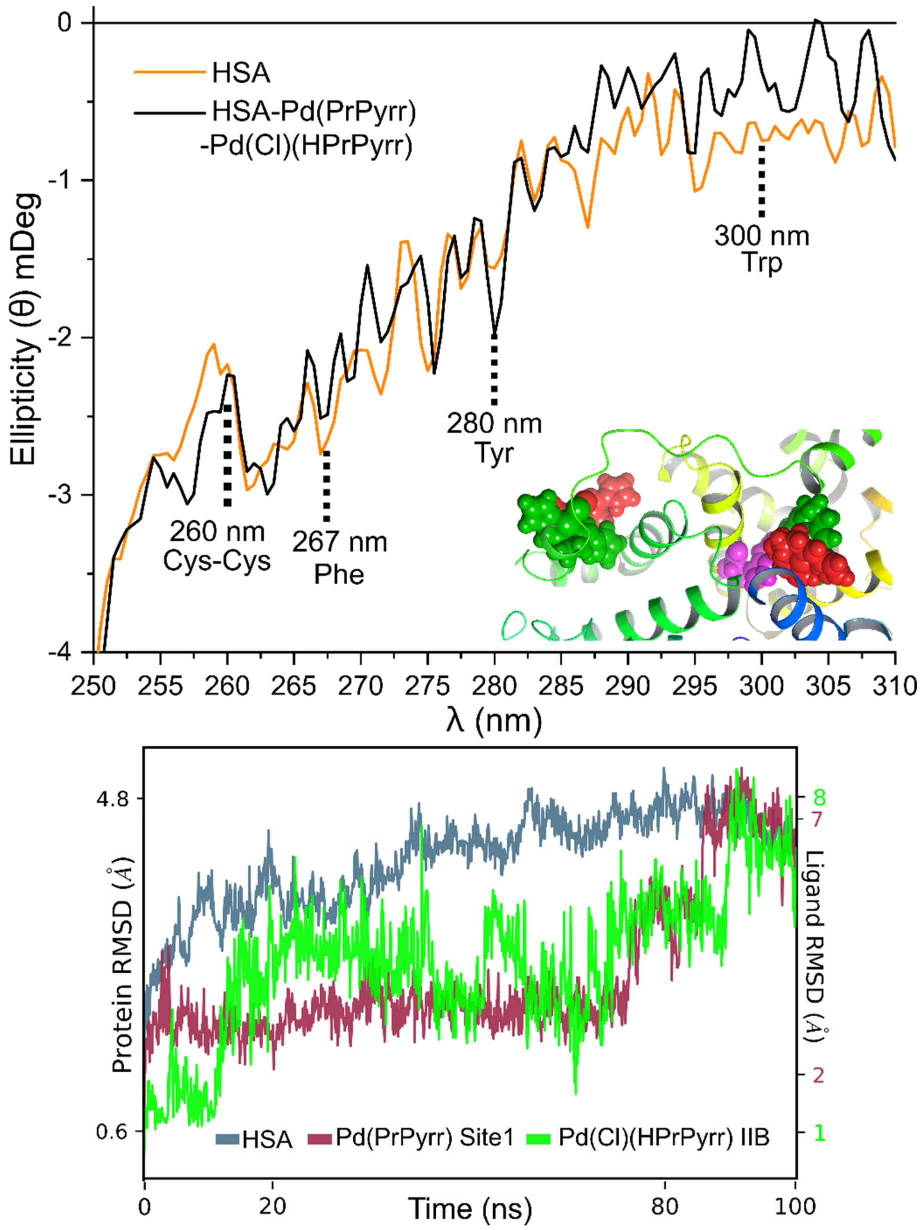

Fig. S15 Top: Near UV-CD fingerprint region of HSA and HSA•{Pd(PrPyrr)}•{Pd(Cl)(HPrPyrr)} with 1 equivalent of Pd^{II}(X) at pH 4. The inset shows Pd(PrPyrr) bound to Site 1 and Pd(Cl)(HPrPyrr) bound at subdomain IIB before (red) and after (green) 100 ns molecular dynamics equilibration. Trp214 is shown in magenta. Bottom: RMSD of HSA and Pd(PrPyrr) docked at Site 1 and Pd(Cl)(HPrPyrr) docked at subdomain IIB over 100 ns. The DFT ICD spectra were calculated before and after MD equilibration with the structure at 0 ns (red) producing the best correlation. The UV-CD shows perturbations at Cys-Cys which are likely due to Pd(PrPyrr) bound at Site 1 within 5 Å of Cys437-Cys448. Additionally, while Trp214 is perturbed by Pd(PrPyrr) at pH 7 and pH 9, the disturbance of this heterocyclic chromophore is greatest at pH 4 and reflects the distance between Pd(PrPyrr) and Trp214 (3 Å at pH 4, 7 Å at pH 7 and 4 Å at pH 9). Phe and Tyr are also significantly perturbed when Pd^{II}(X) is present, indicating the binding site is rich with these aromatic chromophores. Pd(Cl)(HPrPyrr) docked in subdomain IIB is within 5 Å of Phe228, Phe309, Phe326 and Tyr332 while Pd(PrPyrr) docked at Site 1 is stabilized by hydrophobic interactions to Tyr452 resulting in significant disturbance at 280 nm in the near UV-CD spectrum.

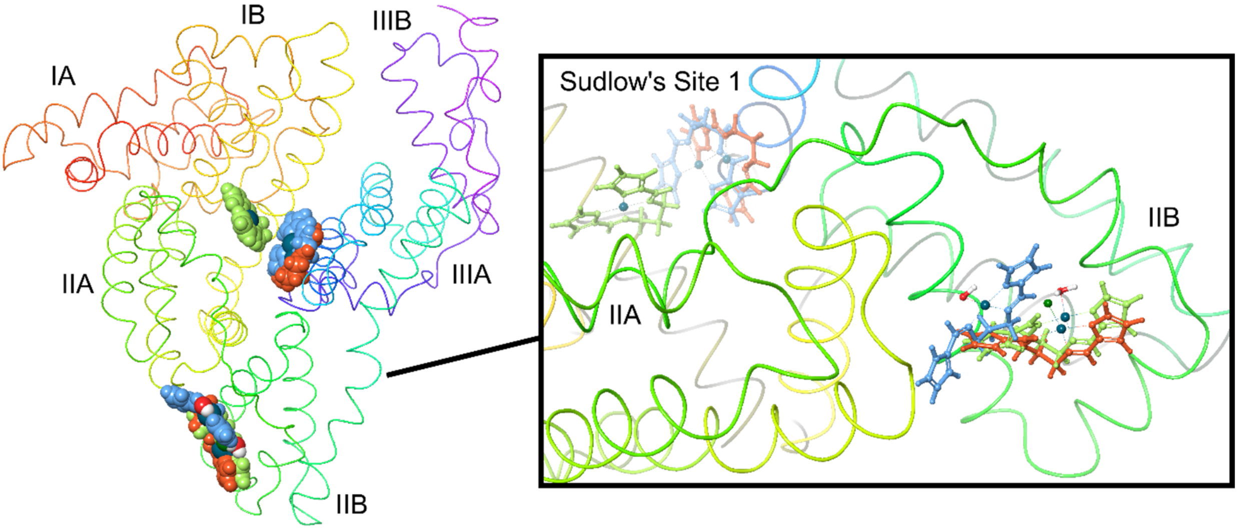

Fig. S16 HSA docked with Pd^{II}(X) at pH 4, 7 and 9. Pd(PrPyrr) docked at pH 4 shown in orange, pH 7 shown in green, and pH 9 shown in blue, the Pd^{2+} ion is shown as metallic-blue. The species Pd(Cl)(HPrPyrr), $[Pd(H_2O)(HPrPyrr)]^+$, and Pd(OH)(HPrPyrr) are also shown with the chloro, aqua, and hydroxyl ligands represented with standard colors of green, red, and white. At all pH's the Pd(PrPyrr) chelate targets Sudlow's Site 1 and the products of hydrolysis target subdomain IIB.

2. Tables

Table. S1 Stern-Volmer quenching constants (K_{SV}), bimolecular quenching rate constants (K_q), for the interaction of Ni^{II}(X) and Pd^{II}(X) with HSA at different temperatures in AMT buffer at pH's 4, 7 and 9.

		Ni^{II}(X)		Pd^{II}(X)	
pH	Temperature (K)	10^{-5} K_{SV} $[M^{-1}]^{a,c}$	K_q [b]	10^{-5} K_{SV} $[M^{-1}]^{a,c}$	K_q [b]
4	288	0.90 (0.02)	1.52E+13	2.24 (0.04)	3.82E+13
	298	0.87 (0.01)	1.47E+13	1.66 (0.03)	2.83E+13
	310	0.52 (0.10)	8.87E+12	0.52 (0.02)	8.91E+12
7	288	1.40 (0.08)	2.38E+13	3.11 (0.2)	5.3E+13
	298	0.87 (0.07)	1.47E+13	2.30 (0.2)	3.92E+13
	310	0.42 (0.01)	7.12E+12	1.71 (0.04)	2.91E+13
9	288	5.00	8.51E+13	3.87 (0.3)	6.6E+13
	298	3.91 (0.03)	6.66E+13	2.70 (0.1)	4.6E+13
	310	2.10 (0.01)	3.58E+13	1.88 (0.2)	3.21E+13

[a] K_{SV} values (Stern-Volmer constants) were determined from fitting the data to Equation (5). [b] Using Equation (6), a mean excited state lifetime, τ, of 5.87(76) ns for HSA was used to calculate the bimolecular quenching rate constants, k_q. [c] The estimated standard deviations of three independent experiments are given in parentheses.
